# Assessing Genetic Diversity and Population Differentiation of Colored Calla Lily (*Zantedeschia* Hybrid) for an Efficient Breeding Program

**DOI:** 10.3390/genes8060168

**Published:** 2017-06-21

**Authors:** Zunzheng Wei, Huali Zhang, Yi Wang, Youli Li, Min Xiong, Xian Wang, Di Zhou

**Affiliations:** 1 Beijing Vegetable Research Center, Beijing Academy of Agriculture and Forestry Sciences; Key Laboratory of Biology and Genetic Improvement of Horticultural Crops, Ministry of Agriculture; Key Laboratory of Urban Agriculture, Ministry of Agriculture, Beijing 100097, China; weizunzheng@163.com (Z.W.); wangyibvrc@163.com (Y.W.); xiongminbvrc@163.com (M.X.); wangxianbvrc@163.com (X.W.); 2 Beijing Institute of Landscape and Garden, Beijing 100102, China; lilytalk2002@163.com; 3 College of Horticulture Science and Technology, Hebei Normal University of Science & Technology, Qinhuangdao 066600, China; 4 Beijing Research Centre of Intelligent Equipment for Agriculture, Beijing 100097, China; shanggaoke@163.com

**Keywords:** *Zantedeschia* species, white calla lily, colored calla lily, EST-SSRs, genetic diversity, population differentiation

## Abstract

Plastome-genome incompatibility (PGI) is prevalent in several plants including the *Zantedeschia* species, a worldwide commercial flower crop native to South Africa. Generally, hybrids suffering from PGI appear less vigorous and more susceptible than normal plants. Previous reports revealed that the PGI level in interspecific hybrids is correlated with the relatedness of the parental species in the genus *Zantedeschia*. To provide a basis for utilizing and improving resources in breeding programs, a total of 117 accessions of colored calla lily (*Zantedeschia* hybrid), collected from New Zealand, the Netherlands and the United States, were genotyped using 31 transferable expressed sequence tags-simple sequence repeats (EST-SSR) markers from the white calla lily (*Zantedeschia aethiopica*). A moderately high level of genetic diversity was observed, with 111 alleles in total, an observed/expected heterozygosity (*Ho/He*) of 0.453/0.478, and polymorphism information content (*PIC*) of 0.26. Genetic distance and STRUCTURE-based analysis further clustered all accessions into four subgroups (G-Ia, G-Ib, G-IIa and G-IIb), which mostly consisted of *Zantedeschia pentlandii, Zantedeschia elliotiana*, *Zantedeschia albomaculata* and *Zantedeschia rehmannii*, respectively. Significant genetic differentiation was observed between all inferred subgroup pairs, with the *Fst* ranging from 0.142 to 0.281. Finally, the accessions assigned into G-IIb (*Z. rehmannii*) were recommended as top priority parents in efficient *Zantedeschia* breeding program designs.

## 1. Introduction

The *Zantedeschia* species of the genus *Zantedeschia* in the family Araceae is a herbaceous perennial flower plant native to the swampy or mountainous regions of South Africa [1,2]. Due to its extraordinary flower spathe (outer “petal” shaped like a funnel) and decorative leaves, the *Zantedeschia* spp. are popular bulb flower crops worldwide. It is generally classified into two sections: the *Zantedeschia* section (white calla lily) and *Aestivea* section (colored calla lily). The former consists of two species (*Zatedeschia aethiopica* Spreng. and *Zantedeschia odorata* Perry.) with a white spathe and rhizome storage organs, while the latter contains six species (*Zantedeschia albomaculata* Baill., *Zantedeschia elliottiana* Engl., *Zantedeschia jucunda* Letty., *Zantedeschia pentlandii* Wittm., *Zantedeschia rehmannii* Engl., and *Zantedeschia valida* Singh.) with a colored spathe and tuberous storage organs [2,3]. In conventional interspecific hybridization between the two sections, hybrids cannot be generated and developed because of endosperm degeneration, abnormal embryo growth and arrested plastid development, which is a known effect of plastome-genome incompatibility (PGI) [4].

Colored calla lilies, also referred to as *Zantedeschia* hybrids, are interspecies hybrids, mainly derived from *Z. elliotiana*, *Z. pentlandii*, *Z. albomaculata* and *Z. rehmannii* within the *Aestivea* section [5]. To date, hundreds of cultivars have been specifically bred for small, medium and large pot production as well as for cut flowers, landscape and garden use in New Zealand, the Netherlands and the United States. As crucial economic flower crops, the export of flowers and tubers of colored calla lily in New Zealand ranked within the top four over the past 23 years, with a value range from NZ$1.9–7.7 million and NZ$0.5–5.4 million, respectively [6]. It also contributed to a substantial proportion of horticultural export earnings in the Netherlands and America. The colored calla lily tuber yield in these two countries has significantly increased in recent years to fulfill foreign market demands in Asia. Yunnan, a southern province in China, is one of the major tuber-importing areas from those three countries. It presently supplies approximately 7.0–10 million flower stems per year, accounting for 15–25% of the global yield. Selection for elite germplasms from natural or hybrid populations are major types of breeding programs in colored calla lily. However, few Chinese organizations or companies have initiated planned colored calla lily improvement programs compared to New Zealand, the Netherlands and the USA. To date, less than ten Chinese varieties have been newly bred through hybridization breeding and released to the public since 1990. One reason is the ill-defined genetic background of the materials used in breeding practices. Another is that biparental inheritance of plastids and PGI are prevalent among interspecific hybrids of the *Aestivae* section [7,8]. The plants that suffer from PGI appear less vigorous and more susceptible than normal plants in same families [4,8]. Genetic diversity assessment is an essential component of germplasm characterization and conservation to identify potential parents for a breeding program [9]. Therefore, it is crucial to clarify the genetic relationships and population structure among colored calla lily germplasms.

The use of molecular markers has become a very powerful tool for the management and utilization of crop genetic diversity in germplasm collections. For *Zantedeschia* spp., some marker types such as inter-simple sequence repeat (ISSR), amplified fragment length polymorphism (AFLP) and random amplified polymorphic DNA (RAPD) have been widely applied in establishing a DNA fingerprint for cultivar identification [10,11], quantifying levels of genetic variation and relatedness [12,13,14], and describing gene flow and parentage in germplasm accessions [15]. Although these molecular markers have proven useful, one major weakness is that they are dominant markers that cannot distinguish heterozygous genotypes from homozygous genotypes [16]. Moreover, these marker systems are labor-intensive, with unstable reproducibility. Simple sequence repeat (SSR) markers possess several advantages over the other molecular markers, including co-dominance, high polymorphism, and good reproducibility [17]. The development of SSR markers can be based on short-length sequences from expressed sequence tags (EST) available at the National Center for Biotechnology Information (NCBI, https://www.ncbi.nlm.nih.gov/) and is feasible for species for which no prior sequence information exists [17,18], including understudied but economically important crops [19]. Fortunately, 209 SSRs loci in 182 assembled EST-unigenes of white calla lily have been identified, 166 of which had flanking sequences suitable for primer design [20]. Here, we first aimed to characterize the frequency and distribution of SSRs and the putative functional annotation of SSR-containing unigenes in detail. Moreover, since the EST sequences represent transcribed regions of the genome, the candidate EST-SSR markers are expected to have high cross-species transferability [17,21]. We then aimed to: (1) assess the cross-species transferability of white calla lily EST-SSRs into colored calla lily; (2) quantify genetic diversity in 117 accessions of colored calla lily from the USA, the Netherlands and New Zealand; (3) detect potential genetic variation and population structure in these colored calla lily accessions; and (4) provide the basis for efficient design of *Zantedeschia* breeding programs in the future.

## 2. Materials and Methods

### 2.1. Plant Material and DNA Extraction

A total of 117 cultivars or hybrids of colored calla lily, including 30 from the USA, 35 from the Netherlands and 52 from New Zealand, have been successively collected by our team since 2004. They were reserved in an experimental greenhouse in the Bulb and Perennial Flowers Genebank collection, Vegetable Research Center, Beijing Academy of Agriculture and Forestry Sciences (Beijing, China). An overview of all colored calla lily accessions is listed in Table S1. Fresh young leaves were collected from all samples in spring 2013 and 2014. Total genomic DNA was extracted using the DNeasy Plant Mini Kit (Zexing Biotech, Beijing, China) following the manufacturer’s protocol. DNA quality and quantification were assessed by electrophoresis on 1.0% (w/v) agarose gels.

### 2.2. SSR Statistics and Distribution, and Functional Annotation of SSR-Containing Unigenes

Wei et al. [20] previously reported that a total of 2175 unigenes including 818 contigs and 1357 singletons were generated from the available 4283 *Z. aethiopica* EST sequences after trimming and eliminating redundancy. As shown in Table 1, the assembled sequences totaled 1.52 Mb; the length of contigs and singlets ranged from 53 to 2073 bp with an average of 704 bp. A total of 209 SSRs loci in 182 unigenes have been identified from the 2175 non-redundant EST sequences of white calla lily. The detailed SSR features including number, frequency and distribution of each motif type were then summarized and characterized in the present study.

Furthermore, to assess the putative functional determination of 182 unigenes containing microsatellite loci, BLASTX searches were performed against the GenBank non-redundant (Nr) protein database (http://blast.ncbi.nlm.nih.gov/Blast.cgi) with an E value cutoff ≤10^−3^. The Blast2GO online platform (http://www.blast2go.org/) was used to assign GO (Gene Ontology) terms, KEGG (Kyoto Encyclopedia of Genes and Genomes) maps and enzyme classification numbers (EC number). In addition, EST-SSR locations in relation to the open reading frame (ORF) were also determined using the ORF Finder program (http://www.ncbi.nlm.nih.gov/gorf/gorf.html).

### 2.3. EST-SSR Marker Cross-Species Transferability and Verification in Colored Calla Lily Accessions

Of all 209 SSRs loci, 166 loci with appropriate flanking sequences [20] were retained for designing primers. The previous experimental validation [20] indicated that 68 EST-SSR primer pairs could yield amenable and reproducible amplicons in 24 *Z. aethiopica* accessions. Therefore, these 68 EST-SSR markers were used to evaluate cross-species transferability and polymorphisms in a panel consisting of 12 randomly selected varieties of colored calla lily. The SSR amplification reactions were conducted as previously described by Wei et al. [20,22,23]. PCR was carried out in the GeneAmp PCR System 9700 (Applied Biosystems, Foster City, CA, USA) with a total volume of 15 μL, containing 1.5 μL 10 × PCR buffer, 30 ng template DNA, 1.2 mM MgCl_2_, 0.8 μL dNTPs (2.5 mM each), 0.4 μL of each primer (10 μM), and 0.25 U TaqDNA polymerase (Tiangen Biotech, Beijing, China). The thermal profile used for amplifications consisted of 5 min of initial denaturation at 95°C, followed by 25 cycles of 30 s at 95 °C, 45 s of annealing at the optimized annealing temperature (Ta) (Table S2), 60 s of extension at 72°C, and a final extension of 20 min at 72 °C. Amplified products were resolved on 8.0% non-denatured polyacrylamide gels and visualized by silver staining [20,22,23]. Fragment sizes were determined by comparison with a 34- to 501-bp pUC19/MspI DNA marker (Zexing Biotech).

### 2.4. Data Analysis

First, the presence of null alleles was tested using Micro-Checker 2.2.3 [24]. Allele frequencies and genetic diversity measures were then estimated by the number of alleles per locus (*Na*), the effective number of alleles (*Ne*), the observed and expected heterozygosity (*Ho* and *He*), polymorphism index content (*PIC*), and private alleles. All calculations were carried out for markers, accessions and categories (origins; *K* = 2 and 4 based on STRUCTURE results) using GENALEX 6.5 [25] and PowerMarker 3.25 [26]. Pairwise *F_ST_* between accessions in the different countries or categories were also calculated using GenAlEx 6.5. To reveal the genetic structure, the Bayesian model-based clustering method implemented in STRUCTURE v2.3 [27] was used to test for the existence of distinct genetic groups and potential admixture among them within the pooled set of individual multi-locus genotypes as previously described. The length of the burn-in period and number of Markov chain Monte Carlo (MCMC) iterations after burn-in were set to 10^5^ and 10^6^, respectively. Algorithm simulation was run ten times for each cluster (*K*), ranging from *K* = 1 to *K* = 10. The most likely *K*-value fitting the data set was assessed using Δ*K* following Evanno et al. [28]. Then, analysis of molecular variance (AMOVA) implemented in GENALEX 6.5 [25] was performed to assess population differentiation among different sub-groups, with 1000 permutations for testing variance components. In addition, genetic relationships among accessions were explored in Power Marker Version 3.25 [26] using the neighbor-joining (NJ) method based on the Nei’s genetic distances. Finally, a NJ tree was generated and visualized using MEGA version 5.0 [29].

## 3. Results and Discussion

### 3.1. Features of SSRs from White Calla Lily EST-Unigenes

As shown in Table 1, approximately 182 (8.37%) of the assembled unigenes harbored 209 SSR motifs, suggesting an average frequency of approximately 1 SSR per 7.27 kb and per 10.40 unique unigenes. In terms of numbers of SSRs per identified EST in our dataset, 88.2% (160) of ESTs had only one SSR while two, three and four SSRs were present in 9.63% (18), 1.60% (3) and 0.55% (1) of ESTs, respectively. Recently, a total of 7997 (20.3%) and 10,089 (12.45%) SSR loci containing 2–6 bp repeat motifs were identified from 39,298 and 81,072 de novo unigenes of *Z. rehmannii* [22] and *Z. aethiopica* [23,30], respectively. The abundance of SSRs in *Z. rehmannii* and *Z. aethiopica* were estimated to be one SSR locus per 4.1 kb and 6.7 kb. Obviously, the frequency of EST-SSR (one SSR per 7.27 kb) in the present study was comparable to that found in *Z. aethiopica*, but lower than that reported in *Z. rehmannii*. Different search criteria and the size of dataset are the main reasons for the highly variable in distribution and frequency of EST-SSRs [22,23,31].

**Figure 1:**
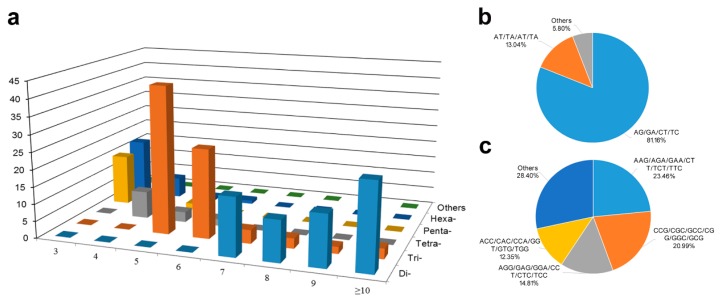
Summary of the simple sequence repeats (SSRs) distribution (**a**), the main motif types of dinucleotide repeats (**b**) and trinucleotide repeats (**c**) in assembled unigenes of white calla lily.

Significant heterogeneity was also observed in microsatellite repeat types and distribution in white calla lily (Figure 1). As shown in Figure 1a, the compilation of all SSRs revealed that tri-nucleotide repeats (81, 38.76%) were the most abundant repeat type, closely followed by di-nucleotide repeats (69, 33.01%), whereas tetra-, penta-, hexa-, and hepta-nucleotide repeats represented approximately 28.23% (59). Among the dinucleotide repeats (Figure 1b), the AG/GC/CT/TC motifs were the most frequent (59, 81.16%), followed by the AT/TA /AT/TA motifs (9, 13.04%). CG/CG (1, 1.45%) were so rare that they almost could not be identified. Among the trinucleotide repeats (Figure 1c), AAG/AGA/GAA/CTT/TCT/TTC motifs (19) and CCG/CGC/GCC/CGG/GGC/GCG (17) were the most common, accounting for 23.46% and 20.99%, followed by AGG/GAG/GGA/CCT/CTC/TCC (12, 14.81%) and ACC/CAC/CCA/GGT/GTG/TGG (10, 12.35%). The microsatellite motifs AG/CT and AGG/CCT in de novo *Z. rehmannii* and *Z. aethiopica* transcripts [22,23] were also identified as predominant in dimeric and trimeric repeat motifs, respectively. Other motifs were identified in insignificant numbers. The SSR motifs were also assessed for their repetitive unit length. The SSR length (motif length × repeat numbers) varied from 12 to 50 bases. Most of them ranged from 12 to 20 bp, accounting for 78.47% of total SSRs. The maximum repeat number of SSR motifs (AG/GA/TC/CT) reached 25. Five tandem repeats (49, 23.44%) were the most common, followed by three and six tandem repeats (33, 15.79% and 29, 13.88%).

Previous reports revealed that microsatellite loci in transcriptome unigenes are usually more prevalent in the untranslated region (UTR) than the open reading frame (ORF) regions. However, the topography of SSR locations in relation to ORF showed that 53.59% (112) of the SSR sequences were within ORF sequences, while 31.10% (65) and 15.31% (32) were within 5′ UTR and 3′ UTR (Table S2). The relative prevalence of microsatellites in ORFs was not consistent with the recent results of *Zantedeschia* transcriptome surveys [22,23]. This unexpected result might be attributed into an overestimation of the putative coding region length identified by the ORF Finder program [17,32].

### 3.2. Functional Annotation of White Calla Lily ESTs Containing SSR Loci

To extract as much functional information as possible, 182 white calla lily EST-unigenes containing SSR loci were subjected to a BLAST (basic local alignment search tool) search against various databases. Sequence similarity alignments were first compared to the Nr databases using the BLASTx algorithm with an *E*-value threshold of 10^−3^. Of the 182 non-redundant unigenes, 146 showed a significant similarity to known proteins in the Nr database (Table S2), representing the putative function of more than two thirds of the assembled unigenes (80.2%). The remaining unmatched unigenes might be unique to white calla lily. The *E*-value and similarity distributions were also calculated to analyze the BLAST results. Of the mapped unigenes, 81 (55.47%) had annotated proteins (1 × < 10^−50^), and 79 (54.10%) had a BLAST result above the cutoff value and alignment similarity greater than 80% (Figure 1a). In terms of species distribution, the largest number of hits was against *Anthurium amnicola* (95, 65.07%), followed by *Phoenix dactylifera* (6, 4.11%), *Nelumbo nucifera* (5, 3.42%), *Musa acuminata* subsp. *malaccensis* (5, 3.42%), *Elaeis guineensis* (4, 2.74%) and others (31, 21.23%). These results suggested that *Z. aethiopica* and *A. amnicola*, both of which belong to the family Araceae, are more closely related to each other in species-genome structure and evolution.

Based on Nr annotation, 93 unigenes were further assigned one or two GO terms at the third level. The sequences assigned to biological process (160), cellular component (87), and molecular function (170) were categorized into 52 functional groups (Figure 1b). ‘Cellular metabolic process’ (22, 13.75%) and ‘organic substance metabolic process’ (20, 12.50%), ‘ion binding’ (17, 19.54%) and ‘transferase activity’ (12, 13.79%), ‘intracellular’ (39, 22.94%) and ‘intracellular part’ (37, 21.76%) were the dominant GO groups among the three main categories, respectively. However, very few unigenes were found in the clusters ‘macromolecule localization’, ‘autophagy’, ‘enzyme regulator activity’, ‘isomerase activity’, ‘plasma membrane’, ‘endomembrane system’, and ‘extracellular space’, etc. KEGG pathway assignments were also carried out; the mapping results are described in supplemental Table S3. A total of 23 assembled sequences were predicted to be involved in 20 metabolic pathways. The number of sequences ranged from 1 to 2. Only two metabolic pathways, including ‘biosynthesis of antibiotics’ and ‘phenylpropanoid biosynthesis’ were fully represented by two EC numbers.

### 3.3. Cross-Species Transferability and Genetic Diversity of EST-SSR Markers in Colored Calla Lily

Out of the 68 EST-SSR markers [20] that have been successfully amplified in 24 white calla lily accessions, 60 (88.2%) produced a high-quality amplification across the 12 cultivars of colored calla lily, of which 34 (Table S2) were polymorphic with a banding pattern that could be clearly resolved. The genotyping failed for the remaining eight microsatellite loci, likely due to low-quality EST sequences, large introns in the genomic sequence or the presence of polymorphisms within flanking sequences [17,18]. This cross-species/section EST-SSR transferability is generally congruent with our previously published results [23], of which 24 (88.9%) out of 27 EST-SSRs from de novo unigenes of white calla lily were transferable into 16 colored calla lily accessions. The high cross-species amplification of EST-SSR with white calla lily indicated that the sequences containing SSR loci were conserved across species or sections of the *Zantedeschia* genus. Those 34 polymorphic markers included 12 di-, 16 tri-, 2 tetra-, 2 penta- and 2 hexa-nucleotide repeats. ORF prediction revealed that most of these SSR loci were in ORF regions. Eighteen markers in ORFs were polymorphic while thirteen and three were in 5′ UTRs or 3′ UTRs, respectively. Interestingly, more than two-thirds (25) of all polymorphic EST-SSR markers had an associated putative function related to cell development, hormone synthesis and regulation, stress responses or other hypothetical functions.

To explore the genetic diversity available in the genetic collections, 34 polymorphic EST-SSRs were used to genotype a sample of 117 accessions of colored calla lily. Preliminary data analysis using Micro-Checker detected deviations at three loci (ZW019, ZW102, and ZW140) due to null alleles. Therefore, the remaining 31 loci were included in subsequent analyses. As shown in Table 2, a total of 111 alleles were detected across all microsatellite loci. The number of alleles per locus (*Na*) varied between 2 and 10, with an average value of 3.58 alleles. The effective number of alleles (*Ne*) ranged from 1.08 to 4.99, suggesting the occurrence of low frequency alleles in some accessions. The mean observed heterozygosity (*Ho*) was 0.453, ranging from 0.052 to 0.969, whereas the mean expected heterozygosity (*He*) was 0.478, ranging from 0.007 to 0.800. All these polymorphic EST-SSR markers revealed allelic diversity with *PIC* values ranging widely from 0.07 to 0.77 (mean 0.412). This was lower than our previous study (*Na* = 5.23; *Ho* = 0.501; *He* = 0.662; *PIC* = 0.446), which used 43 EST-SSR markers on 24 wild or cultivated accession of white calla lily [20]. Diversity in cultivated colored calla lily is generally reported to be low. This was also observed even when other types of markers such as RAPD and ISSR were used [12,13,14]. This may be related with species habitat environment and cultivation history. Most colored calla lily cultivars are native to cool mountainous regions of South Africa, while white calla lily is native to more tropical regions of the Western Cape of South Africa and not only tolerates, but may flourish in very moist wetland conditions [1,2,3,33,34].

### 3.4. Genetic Relatedness and Population Structure among 117 Colored Calla Lily Accessions

**Figure 2:**
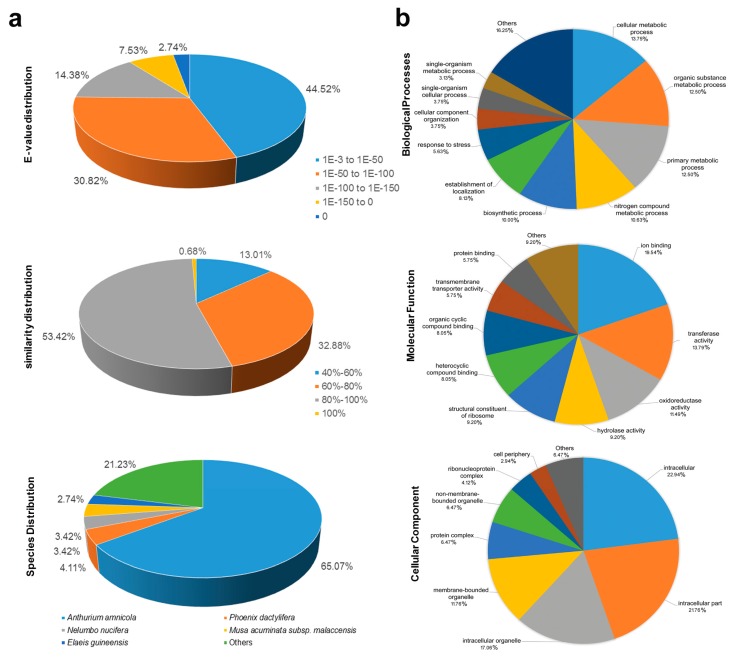
Characteristics of homology search of white calla lily 182 unigenes against the Nr (non-redundant) and GO (Gene Ontology) database. (**a**) The *E*-value, similarity and species distribution of BLAST (basic local alignment search tool) hits for each unique sequence with a cut-off *E*-value of 1 × 10^−3^. (**b**) The GO assignment into biological process, molecular function and cellular component at the third level.

To analyze genetic relationships among accessions, an unweighted NJ tree was constructed based on the EST-SSR genotype data of the 117 colored calla lily accessions. The dendrogram clearly revealed that all accessions could be divided into two main clusters, designated here as Group I (54 accessions) and Group II (63 accessions), respectively (Figure 2a). Each cluster was then further separated into two sub-clusters. Group I-a was mainly comprised of the 20 accessions from the Netherlands (12) and USA (8), whereas Group I-b consisted primarily of 21 New Zealand accessions, followed by 10 Netherlands accessions and 3 USA accessions. Group II-a included 31 accessions, 18 of which were from the Netherlands, 8 from New Zealand, and 5 from the USA. Group II-b contained 32 accessions, among which 14 were from the USA, 11 were from New Zealand, and 7 were from the Netherlands. Apparently, the tree-based clustering in present study did not show distinct associations with the geographic origin of the accessions, suggesting the probable extensive exchange of parental accessions by breeding programs or breeders worldwide. This was also supported by the results inferred by further Bayesian clustering and AMOVA analysis.

A Bayesian-based clustering assignment was also used to determine population structure among 117 colored calla lily accessions using STRUCTURE software. The LnP(D) value increased continuously with the number of clusters (*K*) from 1 to 10 based on estimated posterior probability of genotype data (Figure S1). Therefore, an ad hoc measure Δ*K*, based on the relative rate of change in the likelihood of the data between successive *K* values, was used to determine the optimal number of sub-clusters [28]. The highest Δ*K* values were observed at *K* = 2 (Δ*K* = 23.79) and *K* = 4 (Δ*K* = 6.56), in which all colored calla lily panels were separately classified into two and four subtle sub-groups (Figure S1 and Figure 2b). The membership coefficient (*Q*), a probabilistic assignment of individuals into groups of closely related genetic accessions, is presented in a bar plot (Figure 2b) for each accession compared to the NJ tree. Accessions with *Q* values above 0.90 were assigned to a specific group, whereas accessions with *Q* values lower than 0.90 were categorized as admixture forms. A total of 69 and 63 representatives were assigned to the clusters for *K* = 2 and *K* = 4, respectively; the remaining accessions were considered to be intermediates. The population partition of 117 colored calla lily accessions was largely compatible with NJ clustering results, with very few exceptions.

A vague generalization has attributed most colored calla lily cultivars as interspecies hybrids derived mainly from *Z. elliotiana*, *Z. pentlandii*, *Z. albomaculata* and *Z. rehmannii*. These can be confirmed by our results of NJ tree and STRUCTURE analysis. With *Q* values of ≥0.90, the individuals assigned to sub-populations Group I-a (12 accessions) and I-b (12 accessions) are mainly comprised of Yellow Arums, namely, *Z. pentlandii* and *Z. elliotiana*. The representative accessions in Groups I-a (Gold Affair, Best Gold, Yellow Lemon, Ochre, Inca Gold, Da Huang, Florex Gold, Pot of Gold, Gold Finger, Hot Shot, and Yang Guang) and I-b (B-Y, Black Magic, 9#, Z1, CP-Y-11, BLM, YN, Sunrise, Solid Gold, ZH and Sensation), both have maculate, oblong-hastate to ovate-orbicular leaves with rounded or cordated basal lobes, and a brilliant yellow spathe with or without a purple blotch at the base. Previous research indicated that horticultural hybridization is generally between *Z. pentlandii* and *Z. elliotiana* because of their close relationship in taxonomy [1,2,33]. Many varieties in size, color and leaves have thus been continually produced in breeding programs. Accessions assigned to Group II-a (19) mainly consisted of Black-eyed Arums (*Z. albomaculata*). Black-eyed Arums include two recognized subspecies, *Z. albomaculata* subsp. *macrocarpa* and subsp. *albomaculata* [3]. They are characterized by conspicuously maculate, triangular-hastate to ovate-orbicular-cordate leaves, constricted above triangular-spreading or strap-shaped lobes. Their spathe has a color range from creamy to straw-colored to pale yellow or rarely coral-pink, with limbs more or less truncate. However, we noticed that there is considerable variation in leaf shape and degree of maculation in this group, as well as in spathe shape and color. For example, a substantial proportion of accessions (Captain Camaro, Captain Margarita, Galaxy, Captain Promise, Captain Murano, Captain Chicago, Captain Fuego, Captain Florida, Captain Pairs and Greta) were observed with spathe color ranging from coral-pink to purple, suggesting the complex genetic background of Black-eyed Arums. In fact, the classification of Black-eyed Arums presents one of the greatest problems in the *Zantedeschia* genus [2,3]. A study of plants in their habitats and more extensive herbarium collections showed that a broad view of the species, including *Zantedeschia oculata*, *Zantedeschia hastata*, *Zantedeschia melanoleuca*, *Zantedeschia angustiloba*, *Z. macrocarpa* and *Zantedeschia chloroleuca*, have appeared to be justified for Black-eyed Arums in the past. Therefore, it is very necessary to apply more molecular markers to dissect a potential genetic basis for taxonomic reassessment of Black-eyed Arums in the future. Individuals contained in Group II-b (19) are mainly Pink Arums, namely, *Z. rehmannii*. The representative accessions include Xiangyuan Hong, Rehmannii, Pillow Talk, Mei Yu, Sunglow, Fen B, Wang A, Aurora, Pink Diamond, Rubylite Pink Ice, Hongbaoshi, Saigon Rose, Super Gem, Rubylite Rose, Rose Gem, Amethyst and Lipstick. The striking character that separates these accessions from others is their anceolate or ovate leaves that are cuneate at the base, not lobed [2,3]. Additionally, the pink spathe seems to be most unique. The relationships among the above four inferred sub-groups were consistent with previous results that *Z. elliottiana* and *Z. pentlandii* are distant from the other species, *Z. albomaculata* and *Z. rehmannii*. With species-specific CAPS (cleaved amplified polymorphic sequences) markers developed from the plastidial intergenic region of trnD and trnC, Snijder et al. [8] determined the plastome composition of *Z. aethiopica*, *Z. rehmannii*, *Z. albomaculata* subsp. *albomaculata*, *Z. albomaculata* subsp. *macrocarpa*, *Z. elliottiana* and *Z. pentlandii*. The results showed that *Z. elliottiana* and *Z. pentlandii* displayed a DC-*Alu*I and a DC-*Hae*III restriction pattern that differed from that of *Z. rehmannii* and *Z. albomaculata*.

### 3.5. Genetic Diversity and Population Differentiation within or among Countries and Inferred Groups or Sub-Groups of 117 Accessions of Colored Calla Lily

The allele number, gene diversity and *PIC* were calculated to estimate the genetic diversity in each country, inferred group and sub-group. As shown in Table 3, minor differences in genetic diversity were observed at the country scale. New Zealand exhibited the highest value for most of the indicators (*Na* = 3.226, *Ne* = 2.157, *Ho* = 0.470, *He* = 0.471, and *PIC* = 0.404), followed by the Netherlands and the USA (*Na* = 3.194, *Ne* = 2.104, *Ho* = 0.478, *He* = 0.459, and *PIC* = 0.396; *Na* = 3.161, *Ne* = 2.042, *Ho* = 0.411, *He* = 0.463, and *PIC* = 0.394). It is not unexpected that the most genetic variation revealed by AMOVA (Table 4) was maintained within countries, with a weak but significant genetic differentiation detected among New Zealand, the Netherlands and the USA (*Fst* = 0.021, *p* < 0.001).

The *Zantedeschia* species is known to be native to the African continent and is concentrated mainly in Southern Africa. It has apparently been in cultivation in Europe since the 1660s. The first recorded documentation of *Zantedeschia* species was illustrated in an account of the Royal Garden in Paris in 1664 [33,35]. Introduction of these South African flower crops into the United States of America occurred later, during the second half of the nineteenth century. Currently, the *Zantedeschia* species is a very popular flower at funerals, weddings and practically any festivity in in Western world. Moreover, the globalization of colored calla lily cultivation or production has been initiated from the most represented countries including New Zealand, the Netherlands and the USA. Commercial companies in these countries have expanded their operations offshore by growing flowers and bulbs or even breeding new varieties in China, Kenya, Taiwan, India, and Swaziland for lucrative northern hemisphere markets [36]. Therefore, we believe that genetic diversity differences of colored calla lily among New Zealand, the Netherlands and the USA may be minimized by extensive exchange of germplasm resources. New Zealand is now recognized as a leading world exponent of breeding and growing colored calla lily [36,37]. It actively pioneered the development of longer-lasting, more colorful types and better quality of *Zantedeschia* spathe with longer and stiffer stems. New varieties for both pot and cut flower production continue to be released by *Zantedeschia* breeding programs. Therefore, a relatively large number of accessions was easily collected from New Zealand in the present research. However, we observed that the number of alleles specific to New Zealand (4) and the USA (4) collections are slightly lower than to the Netherlands (6). This is related to the fact that most New Zealand tuber stock originally came from the Netherlands. However, it still needs to be confirmed with further testing and accession sampling. Private alleles are alleles found only in a single population among a broader collection of populations. Those alleles found in our study will be informative for the colored calla lily’s diverse types of population-genetic studies, in such areas as molecular ecology, conservation genetics and especially breeding practices.

**Figure 3:**
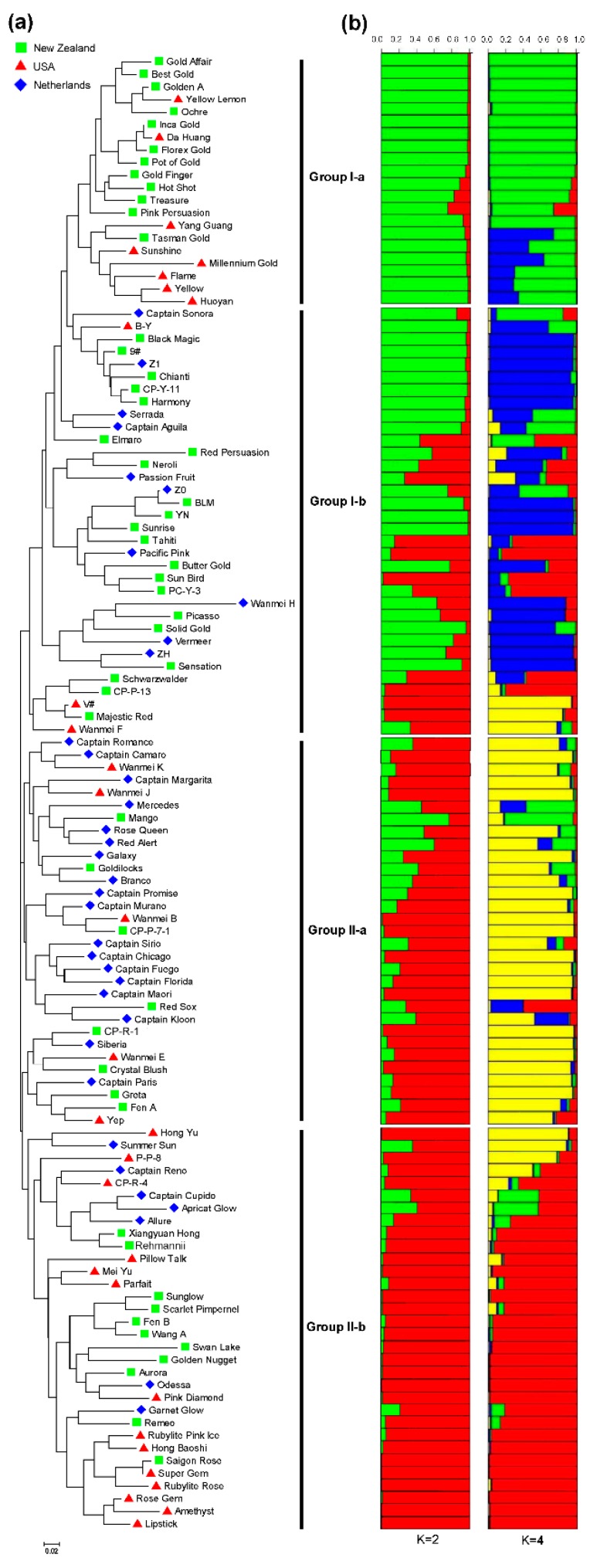
Phylogenic tree aligned with the structure analysis for 117 colored calla lily accessions from New Zealand, the Netherlands and the USA. (**a**) neighbor-joining (NJ) dendrogram based on Nei’s distance of 31 EST-SSR markers showing two major clusters, Group I (54 accessions) and Group II (63 accessions). Each cluster was further separated into two sub-clusters (Group I-a and Group I-b, Group II-a and Group II-b). (**b**) Clustering based on multilocus analysis using the STRUCTURE package. The two best fitting models (*K* = 2 and *K* = 4) according to Evanno’s Δ*K* are shown. Each individual accession is represented by a vertical line divided into *K* colored bars. The length of each colored bar refers to the estimated membership coefficient (*Q*) in different *K* subpopulations for each accession.

The G-I and G-II groups inferred from STRUCTURE analysis contained 31 and 38 accessions, respectively (Table 3). G-II had a higher level of allele richness, gene diversity and *PIC* values (*Na* = 2.839, *Ne* = 1.919, *Ho* = 0.428, *He* = 0.416, and *PIC* = 0.357) than that of G-I (*Na* = 2.654, *Ne* = 1.838, *Ho* = 0.438, *He* = 0.386, and *PIC* = 0.326). Moreover, the G-I group has 17 group-specific alleles while the G-II group has 23 group-specific alleles. The reason for this might be because the G-I group in the present study contained fewer accessions than the G-II group. The G-I and G-II group were each further divided into two sub-groups (G-Ia and G-Ib; G-IIa and G-IIb, respectively). Similar numbers of assigned accessions were observed for each group based on *Q* values of ≥0.90 (Figure 3). Within the G-I and G-II groups, subgroups G-Ib and G-IIa contained the highest number of alleles and effective alleles per locus (*Na* = 2.323 and *Ne* = 1.799; *Na* = 2.548 and *Ne* = 1.847). As mentioned, the individual accessions assigned to the sub-populations Group I-b and II-a are comprised mainly of *Z. elliotiana* and *Z. albomaculata*. The *Z. elliotiana* was assumed to be of hybrid origin because it is known only from cultivated specimens [2,3]. Moreover, the *Z. albomaculata* showed great variation in its morphological characteristics, resulting in the greatest classification problems in the *Zantedeschia* genus. This may be correlated with high level of genetic heterozygosity, group-specific alleles and *PIC* in subgroups G-Ib and G-IIa.

Significant differentiation was observed between the G-I and G-II groups (*Fst* = 0.187) as assessed in AMOVA (Table 4). At the subgroup level, the genetic differentiation among the four inferred sub-populations (G-Ia, G-Ib, G-IIa and G-IIb) was also significant, with an *Fst* of 0.206. Pairwise *Fst* calculations revealed that all sub-groups were significantly different, with the values ranging from 0.142 to 0.281. The lowest genetic differentiation was between G-IIa and G-IIb (*Fst* = 0.142), while the highest genetic differentiation was between G-IIb and G-Ia (*Fst* = 0.281) (Table S4). However, our recent research [23] revealed that the genetic variation was less maintained within the *Zantedeschia* and *Aestivae* sections, but significant genetic differentiation was detected between two sections (*Fst* = 0.69). These may be attributed to incompatibility between the plastome and genome, a general phenomenon among interspecific hybrids between *Zantedeschia* and *Aestivae* sections. Hybrid albinism or chlorophyll deficiency was deemed to be one of the primary post-fertilization barriers to hybridization between two sections in the genus *Zantedeschia* [4]. In fact, interspecific hybrids among the *Aestivae* section also show degrees of chlorophyll deficiency, including albinism. Degrees of PGI were extensive between the hybrid genomes of *Z. rehmannii* and *Z. albomaculata*, *Z. rehmannii* and *Z. elliotiana*, *Z. rehmannii* and *Z. pentlandii* and the plastomes of *Z. albomaculata*, *Z. elliotiana* and *Z. pentlandii*, respectively [7]. Population genetic structure, i.e., non-random spatial distribution of genotypes and alleles, can be shaped by different processes, including restricted gene dispersal, genetic drift, and environmental selection [38]. Thus, it can be expected that the population differentiation among *Zantedeschia* species may be generally influenced by restricted gene dispersal resulting from PGI. The level of plastome genome incompatibility in interspecific hybrids appears to be correlated to the relatedness of the parental species in *Zantedeschia* spp. [7,8]. Based on the directions of PGI and restriction site polymorphisms, Snijder [7] concluded that the plastome of *Z. rehmannii* appeared to be compatible with all tested hybrid genomes in the Aestivae section, and the plastomes of *Z. albomaculata* and *Z. elliotiana* appeared compatible with the hybrid genome of *Z. albomaculata* and *Z. elliotiana*. An investigation of the heredity of resistance to *Erwinia carotovora* subsp. *carotovora* in *Zantedeschia* spp. revealed that *Z. rehmannii* and *Z. albomaculata* contributed more resistance genes than *Z. elliotiana* or *Z. pentlandii* [7]. Bacterial soft rot caused by *E. carotovora* subsp. *carotovora* is a major disease in cultivars of the *Aestivae* section. This soilborne facultative anaerobic pathogen causes maceration and rotting of parenchymatous tissue of all plant organs, eventually resulting in plant death [39,40]. Breeding new resistant cultivars among the *Aestivae* section is always the essential target of breeders worldwide. Therefore, we strongly recommend that the accessions in subgroups G-IIb (*Z. rehmannii*) are given top priority as female or male parents in further efficient design of breeding programs of *Zantedeschia*. Additionally, the accessions in subgroups G-IIa (*Z. albomaculata*) are used as parental plants except when crossing with accessions in G-IIa (*Z. albomaculata*) and/or G-Ib (*Z. elliotiana*).

## 4. Conclusions

Our previous report [20] identified 209 potential SSR loci in 182 assembled EST-unigenes of white calla lily (*Z. aethiopica*). Here, we characterized the frequency, type and distribution of those EST-SSRs in detail. Moreover, most putative functions of 182 SSR-containing unigenes could be assigned by BLAST mapping and GO analysis using the Blast2GO program and KEGG database. Approximately 88.2% (60 out of 68) of EST-SSRs were expected to be transferable to colored calla lily (*Z.* hybrid). Next, 31 polymorphic EST-SSR markers were used to reveal the potential genetic variation and population structure of 117 colored calla lily accessions, which were collected from New Zealand, the Netherlands and the United States. A moderately high level of genetic diversity was observed with *Na* = 3.58, *Ne* = 2.18, *Ho* = 0.453, *He* = 0.478, and *PIC* = 0.412 on average. Genetic distance and model-based analysis further clustered all accessions into two major groups (G-I and G-II) and four subgroups (G-Ia, G-Ib, G-IIa and G-IIb). A higher level of genetic differentiation (*Fst* = 0.187/0.206; *p* < 0.001) was observed among all inferred groups/subgroups than among the three countries (*Fst* = 0.021; *p* < 0.001). These results at the molecular level demonstrate that a substantial portion of colored calla lily accessions were interspecies hybrids derived mainly from *Z. pentlandii, Z. elliotiana*, *Z. albomaculata* and *Z. rehmannii*. PGI are prevalent in *Zantedeschia* species and can result in hybrids that are less vigorous and more susceptible than normal plants. Therefore, the accessions assigned into G-IIb (*Z. rehmannii*) are suggested as top priority parents in *Zantedeschia* conventional breeding. The present findings will facilitate rapid and efficient designs for *Zantedeschia* breeding programs in emerging markets such as China.

## Figures and Tables

**Table 1:** Number and distribution of simple sequence repeats (SSRs) in expressed sequence tags (EST)-unigenes of white calla lily.

Unigenes Features and SSR in Unigenes	Number
Total number of unigenes examined	2175
Number of contigs	818
Number of singletons	1357
Length range of contigs	238–2073 bp
Length range of singletons	53–1095 bp
Total size covered by examined unigenes	1.52 Mb
Total number of SSRs identified	209
Number of unigenes containing one SSR	160
Number of unigenes containing more than one SSR	22
Frequency of SSR occurrence in total number of unigenes	10.40
Frequency of SSR occurrence in total size of unigenes	7.2 kb

**Table 2:** Descriptive statistics of the 31 ESR-SSRs markers scored on 117 colored calla lily accessions.

Loci	*Na*	*Ne*	*Ho*	*He*	*PIC*
ZW008	4	1.43	0.243	0.301	0.29
ZW009	5	2.27	0.969	0.559	0.46
ZW011	6	2.40	0.853	0.584	0.50
ZW012	4	2.45	0.867	0.591	0.52
ZW018	4	2.01	0.895	0.503	0.39
ZW021	2	1.81	0.462	0.448	0.35
ZW029	3	1.61	0.496	0.377	0.31
ZW033	4	2.55	0.405	0.608	0.53
ZW034	5	2.90	0.228	0.655	0.60
ZW037	5	2.42	0.932	0.587	0.50
ZW039	2	1.88	0.231	0.469	0.36
ZW045	5	2.36	0.340	0.575	0.49
ZW046	2	1.85	0.256	0.460	0.35
ZW047	5	3.52	0.402	0.716	0.67
ZW060	2	1.08	0.052	0.070	0.07
ZW062	2	1.90	0.767	0.473	0.36
ZW071	2	1.57	0.474	0.361	0.30
ZW076	5	3.75	0.683	0.733	0.69
ZW080	3	2.94	0.517	0.659	0.59
ZW093	3	2.04	0.164	0.509	0.45
ZW095	3	2.04	0.476	0.511	0.42
ZW096	5	2.61	0.328	0.617	0.57
ZW098	2	1.19	0.172	0.157	0.14
ZW101	2	1.91	0.301	0.476	0.36
ZW122	2	1.17	0.159	0.147	0.14
ZW123	4	1.78	0.549	0.438	0.37
ZW126	10	4.99	0.458	0.800	0.77
ZW132	2	1.31	0.205	0.239	0.21
ZW146	2	1.97	0.389	0.493	0.37
ZW158	2	1.08	0.019	0.072	0.07
ZW165	4	2.72	0.755	0.632	0.56
Mean	3.58	2.18	0.453	0.478	0.412

*Ho*: observed heterozygosity; *He*: expected heterozygosity; *Na*: number of alleles per locus, *Ne*: the effective number of alleles; *PIC*: polymorphism information content.

**Table 3:** Genetic diversity in different populations and sub-populations of colored calla lily.

Source	Population	N	*Na*	*Ne*	*Ho*	*He*	*Private*	*PIC*
Country	Netherlands	34	3.194	2.104	0.478	0.459	6	0.396
	New Zealand	52	3.226	2.157	0.470	0.471	4	0.404
	USA	31	3.161	2.042	0.411	0.463	4	0.394
Model-based population (*K* = 2)	G-I	31	2.654	1.838	0.438	0.386	17	0.326
	G-II	38	2.839	1.919	0.428	0.416	23	0.357
Model-based population (*K* = 4)	G-Ia	12	2.000	1.606	0.394	0.292	1	0.241
	G-Ib	12	2.323	1.799	0.508	0.382	8	0.319
	G-IIa	19	2.548	1.847	0.439	0.390	8	0.327
	G-IIb	19	2.323	1.820	0.437	0.381	4	0.321

**Table 4:** Analysis of molecular variance (AMOVA) between inferred populations of colored calla lily.

	Source	d.f. (degree of freedom)	Sum of Squares	Mean of Squared Observations	Estimation Variance	Percentage of Variations %	Fixation Index
Country	Among countries	2	48.872	24.436	0.184	2%	*Fst* = 0.021
	Among individuals	114	1195.107	10.483	2.022	23%	
	Within individuals	117	753.500	6.440	6.440	74%	
	Total	233	1997.479		8.646	100%	
Model-based population (*K* = 2)	Among groups	1	129.082	129.082	1.757	19%	*Fst* = 0.187
	Within populations	67	610.041	9.105	1.491	16%	
	Among individuals	69	422.500	6.123	6.123	65%	
	Total	137	1161.623		9.371	100%	
Model-based population (*K* = 4)	Among groups	3	192.571	64.190	1.845	21%	*Fst* = 0.206
	Among individuals	58	461.849	7.963	0.860	10%	
	Within individuals	62	387.000	6.242	6.242	70%	
	Total	123	1041.419		8.948	100%	

## Supplementary Materials

The following are available online at www.mdpi.com/2073-4425/8/6/168/s1. Figure S1: Estimates of the posterior probability of the data at a given *K* for 117 colored calla lily accessions. Table S1: List of 117 colored calla lily (*Zantedeschia* hybrid) accessions used for genetic diversity and population structure analysis. Table S2: Characteristics of the 166 EST-SSR markers developed from 182 SSR-containing unigenes of white calla lily. Table S3: KEGG annotation and its corresponding EC number of 182 SSR-containing unigenes of white calla lily. Table S4: Pairwise population *Fst* Values (*p* < 0.001) between inferred four sub-groups of colored calla lily.
