# Supplementary material for: Assessing Genetic Diversity and Population Differentiation of Colored Calla Lily (Zantedeschia Hybrid) for an Efficient Breeding Program
Source: Genes (Basel). 2017 Jun 21;8(6):168. doi: 10.3390/genes8060168 (PMC5485532; doi:10.3390/genes8060168)
Supplement: Supplementary file 1 [file genes-08-00168-s001.zip › Table S1.docx]

**Table S1.** List of 117 colored calla lily (*Zantedeschia* hybrid) accessions used for genetic diversity and population structure analysis.

| **Accessions** | **Country** | **Color** | **Leaves** | **Use type** | **Photographs** |
| --- | --- | --- | --- | --- | --- |
| 9# | New Zealand | Yellow | Saggitate, spotted | Cut-flower | 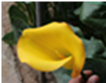 |
| Allure | Netherlands | Purple | Ovate, spotted | Pot-flower | 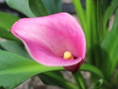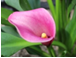 |
| Amethyst | USA | Purple | Ovate, spotted | Pot-flower | 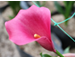 |
| Apricat Glow | Netherlands | Orange | Hastate, not spotted | Pot-flower | 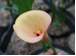 |
| Aurora | New Zealand | Pink | Lanceolate, spotted | Pot-/Cut-flower | 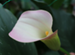 |
| Best Gold | New Zealand | Yellow | Saggitate, spotted | Cut-flower | 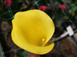 |
| Black Magic | New Zealand | Yellow | Saggitat, spotted | Cut-flower | 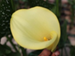 |
| BLM | New Zealand | Yellow | Saggitate, spotted | Cut-flower | 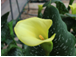 |
| Branco | Netherlands | White | Hastate, spotted | Pot-flower | 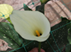 |
| Butter Gold | New Zealand | Yellow | Saggitate, spotted | Cut-flower | 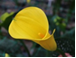 |
| B-Y | USA | Yellow | Saggitate, spotted | Pot flower | 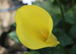 |
| Captain Aguila | Netherlands | Yellow | Saggitate, spotted | Pot-/Cut-flower | 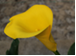 |
| Captain Camaro | Netherlands | Purple | Hastate, spotted | Pot-flower | 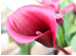 |
| Captain Chicago | Netherlands | Pink | Hastate, spotted | Pot-/Cut-flower | 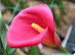 |
| Captain Cupido | Netherlands | white | Hastate, not spotted | Pot-flower | 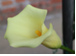 |
| Captain Florida | Netherlands | Red | Hastate, spotted | Pot-/Cut-flower | 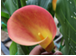 |
| Captain Fuego | Netherlands | Orange | Hastate, spotted | Pot-/Cut-flower | 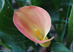 |
| Captain Kloon | Netherlands | Yellow | Hastate, spotted | Pot-/Cut-flower | 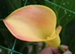 |
| Captain Maori | Netherlands | Purple | Saggitate, spotted | Pot-/Cut-flower | 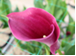 |
| Captain Margarita | Netherlands | White | Ovate, spotted | Pot-/Cut-flower | 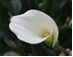 |
| Captain Murano | Netherlands | Orange | Ovate, spotted | Pot-/Cut-flower | 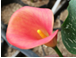 |
| Captain Paris | Netherlands | Purple | Hastate, spotted | Pot-/Cut-flower | 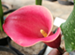 |
| Captain Promise | Netherlands | Purple | Saggitate, spotted | Pot-/Cut-flower | 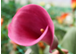 |
| Captain Reno | Netherlands | Purple | Hastate, spotted | Pot-/Cut-flower | 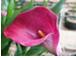 |
| Captain Romance | Netherlands | Pink | Hastate, spotted | Pot-/Cut-flower | 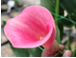 |
| Captain Sirio | Netherlands | Orange | Hastate, spotted | Pot-/Cut-flower | 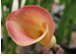 |
| Captain Sonora | Netherlands | Yellow | Saggitate, spotted | Pot-/Cut-flower | 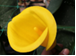 |
| Chianti | New Zealand | Pink | Ovate, spotted | Pot-/Cut-flower | 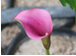 |
| CP-P-13 | New Zealand | Pink | Ovate, spotted | Pot-/Cut-flower | 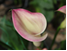 |
| CP-P-7-1 | New Zealand | Red | Ovate, spotted | Pot-/Cut-flower | 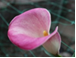 |
| CP-R-1 | New Zealand | Red | Lanceolate, not spotted | Pot-/Cut-flower | 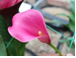 |
| CP-R-4 | USA | Purple | Ovate, spotted | Pot-/Cut-flower | 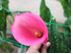 |
| CP-Y-11 | New Zealand | Yellow | Saggitate, spotted | Cut-flower | 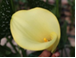 |
| Crystal Blush | New Zealand | Pink | Lanceolate, not spotted | Pot-/Cut-flower | 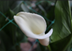 |
| Da Huang | USA | Yellow | Saggitate, spotted | Pot-/Cut-flower | 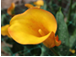 |
| Elmaro | New Zealand | Orange | Saggitate, spotted | Pot-/Cut-flower | 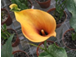 |
| Fen A | New Zealand | Pink | Ovate, spotted | Pot-/Cut-flower | 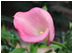 |
| Fen B | New Zealand | Pink | Ovate, not spotted | Pot-/Cut-flower | 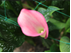 |
| Flame | USA | Orange | Saggitate, spotted | Pot-/Cut-flower | 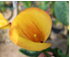 |
| Florex Gold | New Zealand | Yellow | Hastate, spotted | Cut-flower | 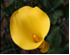 |
| Galaxy | Netherlands | Red | Hastate, spotted | Pot-/Cut-flower | 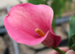 |
| Garnet Glow | Netherlands | Red | Lanceolate, not spotted | Pot-flower | 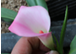 |
| Gold Affair | New Zealand | Yellow | Hastate, spotted | Pot-/Cut-flower | 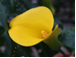 |
| Gold Finger | New Zealand | Yellow | Saggitate, spotted | Pot-/Cut-flower | 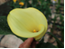 |
| Golden A | New Zealand | Yellow | Hastate, spotted | Pot-/Cut-flower | 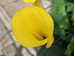 |
| Golden Nugget | New Zealand | Yellow | Ovate, spotted | Pot-/Cut-flower | 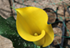 |
| Goldilocks | New Zealand | Yellow | Ovate, spotted | Pot-flower | 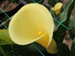 |
| Greta | New Zealand | Purple | Ovate, spotted | Pot-flower | 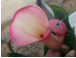 |
| Harmony | New Zealand | White | Ovate, spotted | Pot-/Cut-flower | 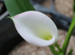 |
| Hong Baoshi | USA | Pink | Lanceolate, not spotted | Pot-flower | 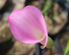 |
| Hong Yu | USA | Purple | Ovate, not spotted | Pot-flower | 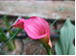 |
| Hot Shot | New Zealand | Orange | Hastate, spotted | Cut-flower | 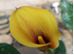 |
| Huoyan | USA | Yellow | Saggitate, spotted | Pot-flower | 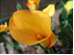 |
| Inca Gold | New Zealand | Yellow | Saggitate, spotted | Pot-/Cut-flower | 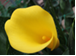 |
| Lipstick | USA | Pink | Lanceolate, not spotted | Pot-flower | 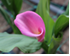 |
| Majestic Red | New Zealand | Red | Lanceolate, not spotted | Pot-/Cut-flower | 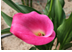 |
| Mango | New Zealand | Orange | Hastate, spotted | Pot-/Cut-flower | 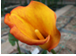 |
| Mei Yu | USA | Purple | Lanceolate, not spotted | Pot-flower | 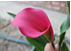 |
| Mercedes | Netherlands | Orange | Ovate, not spotted | Pot-flower | 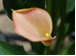 |
| Millennium Gold | USA | Yellow | Saggitate, spotted | Pot-/Cut-flower | 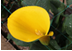 |
| Neroli | New Zealand | Orange | Hastate, spotted | Pot-/Cut-flower | 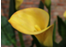 |
| Ochre | New Zealand | Orange/Yellow | Saggitate, spotted | Pot-/Cut-flower | 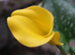 |
| Odessa | Netherlands | Purple | Ovate, spotted | Pot-flower | 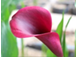 |
| Pacific Pink | New Zealand | Pink | Hastate, spotted | Cut-flower | 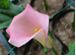 |
| Parfait | USA | Pink | Ovate, spotted | Pot-/Cut-flower | 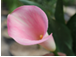 |
| Passion Fruit | Netherlands | Orange | Hastate, spotted | Pot-/Cut-flower | 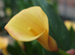 |
| PC-Y-3 | New Zealand | Orange | Hastate, spotted | Pot-/Cut-flower | 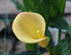 |
| Picasso | New Zealand | Purple | Saggitate, spotted | Pot-/Cut-flower | 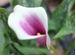 |
| Pillow Talk | USA | Pink | Lanceolate, not spotted | Pot-/Cut-flower | 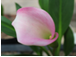 |
| Pink Diamond | USA | Pink | Lanceolate, not spotted | Pot-/Cut-flower | 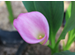 |
| Pink Persuasion | New Zealand | Pink | Ovate, spotted | Pot-/Cut-flower | 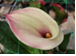 |
| Pot of Gold | New Zealand | Yellow | Saggitate, spotted | Pot-/Cut-flower | 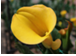 |
| P-P-8 | USA | Pink | Ovate, spotted | Pot-flower | 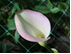 |
| Red Alert | Netherlands | Orange | Saggitate, spotted | Pot-flower | 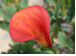 |
| Red Persuasion | New Zealand | Red | Ovate, spotted | Pot-/Cut-flower | 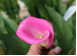 |
| Red Sox | New Zealand | Orange | Hastate, spotted | Pot-/Cut-flower | 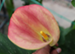 |
| Rehmannii | Netherlands | Pink | Lanceolate, not spotted | Pot-flower | 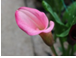 |
| Remeo | New Zealand | Pink | Ovate, spotted | Pot-/Cut-flower | 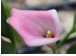 |
| Rose Gem | USA | Pink | Lanceolate, not spotted | Pot-flower | 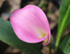 |
| Rose Queen | Netherlands | Pink | Hastate , not spotted | Pot-flower | 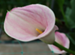 |
| Rubylite Pink Ice | USA | Pink | Ovate, not spotted | Pot-flower | 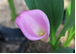 |
| Rubylite Rose | USA | Pink | Lanceolate, not spotted | Pot-/Cut-flower | 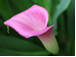 |
| Saigon Rose | New Zealand | Purple | Lanceolate, not spotted | Pot-flower | 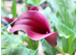 |
| Scarlet Pimpernel | New Zealand | Red | Ovate, spotted | Pot-flower | 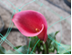 |
| Schwarzwalder | New Zealand | Purple | Ovate, spotted | Pot-/Cut-flower | 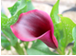 |
| Sensation | New Zealand | Orange | Saggitate, spotted | Pot-/Cut-flower | 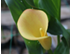 |
| Serrada | Netherlands | Yellow | Saggitate, spotted | Pot-flower | 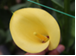 |
| Siberia | Netherlands | White | Ovate, spotted | Pot-/Cut-flower | 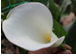 |
| Solid Gold | New Zealand | Yellow | Saggitate, spotted | Cut-flower | 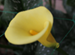 |
| Summer Sun | Netherlands | Yellow | Hastate, spotted | Pot-/Cut-flower | 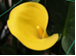 |
| Sun Bird | New Zealand | Yellow | Hastate, spotted | Cut-flower | 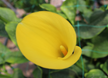 |
| Sunglow | New Zealand | Red | Ovate, spotted | Pot-flower | 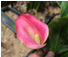 |
| Sunrise | New Zealand | Orange | Saggitate, spotted | Cut-flower | 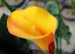 |
| Sunshine | USA | Yellow | Saggitate, spotted | Pot-/Cut-flower | 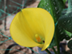 |
| Super Gem | USA | Pink | Lanceolate, not spotted | Pot-flower | 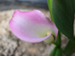 |
| Swan Lake | New Zealand | White | Saggitate, spotted | Pot-/Cut-flower | 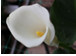 |
| Tahiti | New Zealand | Yellow | Hastate, spotted | Pot-/Cut-flower | 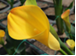 |
| Tasman Gold | New Zealand | Yellow | Saggitate, spotted | Pot-/Cut-flower | 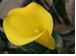 |
| Treasure | New Zealand | Orange | Hastate, spotted | Cut-flower | 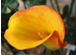 |
| V# | USA | Purple | Hastate, spotted | Pot-flower |  |
| Vermeer | Netherlands | Purple | Saggitate, spotted | Pot-/Cut-flower |  |
| Wang A | New Zealand | Pink | Ovate, spotted | Pot-flower |  |
| Wanmei B | USA | White | Ovate, spotted | Pot-/Cut-flower |  |
| Wanmei E | USA | Pink | Ovate , spotted | Pot-flower |  |
| Wanmei F | USA | White | Ovate, spotted | Pot-/Cut-flower |  |
| Wanmei H | USA | White | Ovate, spotted | Pot-flower |  |
| Wanmei J | USA | White | Ovate, spotted | Pot-flower |  |
| Wanmei K | USA | Pink | Ovate, spotted | Pot-flower |  |
| Xiangyuan Hong | New Zealand | Red | Ovate, spotted | Pot-/Cut-flower |  |
| Yang Guang | USA | Yellow | Saggitate, spotted | Pot-flower |  |
| Yellow | USA | Yellow | Saggitate, spotted | Pot-flower |  |
| Yellow Lemon | USA | Yellow | Saggitate, spotted | Pot-/Cut-flower |  |
| Yep | USA | Pink | Ovate, spotted | Pot-flower |  |
| YN | New Zealand | Yellow | Saggitate, spotted | Cut-flower |  |
| Z0 | Netherlands | Yellow | Saggitate, spotted | Cut-flower |  |
| Z1 | Netherlands | Yellow | Saggitate, spotted | Cut-flower |  |
| ZH | Netherlands | Yellow | Saggitate, spotted | Cut-flower |  |
