# Supplementary material for: Assessing Genetic Diversity and Population Differentiation of Colored Calla Lily (Zantedeschia Hybrid) for an Efficient Breeding Program
Source: Genes (Basel). 2017 Jun 21;8(6):168. doi: 10.3390/genes8060168 (PMC5485532; doi:10.3390/genes8060168)
Supplement: Supplementary file 1 [file genes-08-00168-s001.zip › Table S2.docx]

**Table S3.** Characteristics of the 166 EST-SSR markers developed from 182 SSR-containing unigenes of white calla lily.

| **Locus** | **Accession no.** | **Primers sequence (5′-3′)** | **Motif** | **SSR location** | **Size (bp)** | ***Ta* (℃)** | **Description** | **E value** |
| --- | --- | --- | --- | --- | --- | --- | --- | --- |
| ZW001 | Contig_13 | F: TCTCTTCCCCCCTCTCTCTC  R: ACTCAATCTCCGACTTCCGA | (GCC)7 | 5'UTR | 228 | 55.1 | 60S ribosomal protein L30 [*Anthurium amnicola*] | 1.00E-65 |
| ZW002 | Contig_18 | F: AGCTGGCGGTTAAGATGGT  R: GGTGGTGATGCTGTTATTT | (ACC)9 | ORF | 254 | 54.7 | Translation initiation factor IF-2 [*Anthurium amnicola*] | 4.00E-36 |
| ZW003 | Contig_24 | F: GCCTTCCTGCTGGTTTCGT  R: CGGCGTCAACAGCGATTACA | (GCC)6 | ORF | 292 | 58.8 | CYC02 protein [*Anthurium amnicola*] | 2.00E-05 |
| ZW004 | Contig_33 | F: TGTGGCTGGTTAGTAGAAA  R: TAGTAGGGGAATCCACAAT | (AAAAAG)3 | 5’UTR | 198 | 48.8 | hypothetical protein g.43869 [*Anthurium amnicola*] | 7.00E-15 |
| ZW005 | Contig_33 | F: TCTGCTTTCTTGCTGGACCATT  R: GGTGGTGATGCTGTTATTT | (GA)15 | ORF | 103 | 49.8 | hypothetical protein g.43869 [*Anthurium amnicola*] | 7.00E-15 |
| ZW006 | Contig_46 | F: AGCAGCCACCAACGAAAGA  R: TCGCACCATTGGGAACTTA | (AAAGAA)3 | 5’UTR | 179 | 50.8 | polyprotein precursor [*Zantedeschia mosaic* virus] | 0 |
| ZW007 | Contig_62 | F: GATACTGTGATTCTGTTGGA  R: TGTAGAGTTCAGAGGGTTA | (GGCA)4 | ORF | 191 | 46.4 | None | None |
| ZW008^※^ | Contig_63 | F: GTAGCATAGCACAAGGCAGA  R: CTTTAGGGGAGGCGAGGTT | (GA)9 | 5’UTR | 182 | 55.8 | Auxin-repressed 12. protein [*Anthurium amnicola*] | 1.00E-39 |
| ZW009^※^ | Contig_73 | F: TGATGCTCGGACTCGGAACA  R: AAACAAGACCCCCACGGACA | (AGA)5 | ORF | 180 | 54.4 | None | None |
| ZW010 | Contig_77 | F: TGCGTCTTCTCCAGTTCG  R: TGCTCTTCCAGTAGCGGTT | (CTC)6 | 5’UTR | 192 | 56.3 | 40S ribosomal protein S11 [*Anthurium amnicola*] | 4.00E-87 |
| ZW011^※^ | Contig_99 | F: CACTTTTTTCTTCCACCTG  R: CGCTCCCATTATACTCAA | (CT)9 | 5’UTR | 118 | 50.5 | Proteasome subunit beta type-3 [*Anthurium amnicola*] | 3.00E-144 |
| ZW012^※^ | Contig_105 | F: TCCACTTGCTCTGCTTTGCCA  R: TGCCGCCACCAATAAAGTCAT | (TCCT)5 | 5’UTR | 175 | 56.6 | Microtubule-associated protein 70-2 [*Anthurium amnicola*] | 4.00E-15 |
| ZW013 | Contig_105 | F: GACGCCTTTCCCTCTCCTCTT  R: CCAAATCGGTCTCATTCTCCAT | (CTC)6 | 5’UTR | 174 | 56.1 | Microtubule-associated protein 70-2 [*Anthurium amnicola*] | 4.00E-15 |
| ZW014 | Contig_110 | F: GGAGCACAGGGAGAGCAGAGAT  R: TCCACGCCAGGGAAACGAT | (GA)9 | 5’UTR | 99 | 54.4 | Cathepsin B [*Anthurium amnicola*] | 0 |
| ZW015 | Contig_110 | F: GCTGCTTCTCATATCGTTTCCCT  R: GGCTTGTTGGCATCCTCTT | (CTG)5 | ORF | 140 | 56.3 | Cathepsin B [*Anthurium amnicola*] | 0 |
| ZW016 | Contig_120 | F: TACGCTCTCTCCATACCTGT  R: CCTAAACGAAAAGGAGTACA | (AAAT)7 | 3’UTR | 157 | 43.4 | caffeic acid O-methylltransferase [*Chrysanthemum* x *morifolium*] | 4.00E-62 |
| ZW017 | Contig_121 | F: CTGCTACGCTCTCTCCATA  R: AGGTTTTACGTTCCCAA | (AAAT)7 | 3’UTR | 200 | 45.3 | caffeic acid O-methylltransferase [*Chrysanthemum* x *morifolium*] | 9.00E-16 |
| ZW018^※^ | Contig_144 | F: TCCACCTCGCCGAGCACTTA  R: CAGTCGCACAAGAGGAGAGCA | (GCT)5 | ORF | 113 | 54.4 | None | None |
| ZW019^※^ | Contig_146 | F: CCTCTGCTGTTGCTGCCTT  R: AGGATGGTTCCAAAGCACTA | (GAAAG)3 | ORF | 184 | 52.3 | None | None |
| ZW020 | Contig_151 | F: GCCATACACCTCGTGCTCCT  R: GGGGTTCATTTCCTCCTCGT | (TC)15 | ORF | 173 | 57.2 | Secretory carrier-associated membrane protein 4 [*Anthurium amnicola*] | 5.00E-152 |
| ZW021^※^ | Contig_161 | F: TGGCGACCAAGGAAGTTTA  R: ATGGTGACAAGCACAAACAACA | (AG)9 | ORF | 104 | 50.5 | None | None |
| ZW022 | Contig_166 | F: GCCAGTTTAGGTGGTTCTT  R: CGTTTCATTTCTCTATACATCG | (AG)7 | 3’UTR | 245 | 48.7 | Thiamine pyrophosphokinase [*Anthurium amnicola*] | 5.00E-100 |
| ZW023 | Contig_173 | F: CAGTGGTTGACCGTTATGTA  R: CCTAACCTGAAACTTCTCCA | (TG)7 | 5’UTR | 114 | 47.6 | None | None |
| ZW024 | Contig_179 | F: GCGTCGTAGCAGACCCAGTTTA  R: CAGTCGGCGCTGTTGAAGAGTT | (GGC)5 | ORF | 273 | 59.3 | Antimicrobial peptide 1 [*Anthurium amnicola*] | 3.00E-17 |
| ZW025 | Contig_179 | F: GACATCACAGCCCAGCACT  R: GACCAGAAACAAGGCACTA | (TTC)13 | ORF | 283 | 50.5 | Antimicrobial peptide 1 [*Anthurium amnicola*] | 3.00E-17 |
| ZW026 | Contig_181 | F: CGTCGTAGCAGACCCAGTT  R: CGGTGTAGACAAAGTAGTA | (GGC)5 | ORF | 238 | 52.5 | Antimicrobial peptide 1 [*Anthurium amnicola*] | 3.00E-17 |
| ZW027 | Contig_185 | F: GCTCCTGTATCTCCACCTGT  R: GCCTTCTTTTCCTCCTCTGT | (GAT)5 | ORF | 104 | 50.2 | PREDICTED: elongation factor 1-delta [*Vitis vinifera*] | 2.00E-98 |
| ZW028 | Contig_188 | F: ATTCCGATGGGGAGCAGAGA  R: TTTTGATGCCCAACTGCGA | (CAC)5 | ORF | 177 | 57.5 | Cysteinyl leukotriene receptor 1 [*Anthurium amnicola*] | 3.00E-158 |
| ZW029^※^ | Contig_190 | F: ATGACTTTTCCCCATGACA  R: CAGAACGGAGTGGTTAGAGA | (GCAT)4 | 3’UTR | 115 | 51.0 | Cyclic nucleotide-gated ion channel 4 [*Anthurium amnicola*] | 6.00E-88 |
| ZW030 | Contig_199 | F: GTAGTTCCTTAGGGTACTGGGT  R: ATGAAGACTTGGATGATGCT | (ATG)5 | 5’UTR | 159 | 47.6 | putative aspartic protease At2g35615 [*Anthurium amnicola*] | 3.00E-34 |
| ZW031 | Contig_218 | F: TGAAGAACAAGGACGAGGT  R: CTCTCAAGGCAGGAATGTC | (AGA)5 | ORF | 192 | 53.6 | None | None |
| ZW032 | Contig_229 | F: TGGTTTGCTGTGTAGTGCGA  R: CAAGAAATGGAAGGGGGAGA | (GA)8 | 5’UTR | 176 | 56.9 | putative mannitol dehydrogenase [*Anthurium amnicola*] | 0 |
| ZW033^※^ | Contig_268 | F: CTGCCGTCGTTACTACTACT  R: CTGCTTGATGTGGCTGTCT | (CTA)5 | 3’UTR | 198 | 55.1 | PREDICTED: probable xyloglucan endotransglucosylase/hydrolase protein 6 [N*elumbo nucifera*] | 3.00E-155 |
| ZW034^※^ | Contig_268 | F: AACCACCTCATCTACGACTA  R: ACTTCTTCTGTTCCTCCTGT | (TC)9 | ORF | 175 | 53.8 | PREDICTED: probable xyloglucan endotransglucosylase/hydrolase protein 6 [*Nelumbo nucifera*] | 3.00E-155 |
| ZW035 | Contig_272 | F: AAGACGACCACCAAGACCGA  R: GTTGCCAGCCATCAGCAGAGA | (ACGACA)3 | ORF | 189 | 57.9 | Protein TIFY 3B [*Anthurium amnicola*] | 2.00E-25 |
| ZW036 | Contig_277 | F: GTCGGATTCCGGGTCGA  R: GAAGCTCTGCCTCTGTTGGA | (CTCG)4 | 5’UTR | 234 | 56.9 | PREDICTED: calmodulin-7 isoform X1 [*Vitis vinifera*] | 4.00E-100 |
| ZW037^※^ | Contig_280 | F: GCAAGGGGAAACCAAGCGT  R: GGAGGAGAAGGGCGTAAGA | (GCC)6 | ORF | 102 | 58.4 | Diaminopimelate epimerase, chloroplastic [*Anthurium amnicola*] | 7.00E-145 |
| ZW038 | Contig_282 | F: CCTCCACCCAGAGCAACTA  R: CAAAACAGGCAGATTAAGCA | (ATTAAG)3 | ORF | 144 | 47.7 | PREDICTED: G-type lectin S-receptor-like serine/threonine-protein kinase At2g19130 [*Nelumbo nucifera*] | 3.00E-64 |
| ZW039^※^ | Contig_291 | F: GACGCCTACCTCCTCAAGA  R: CCCCCTCCCCATACATACT | (GCG)6 | 3’UTR | 149 | 53.1 | MLP-like protein 423 [*Anthurium amnicola*] | 2.00E-70 |
| ZW040 | Contig_300 | F: CCTTCCTGCTGGTTTCGTCTGA  R: CTCGGCGTCAACAGCGATTACA | (GCC)6 | ORF | 293 | 60.0 | CYC02 protein [*Anthurium amnicola*] | 2.00E-05 |
| ZW041 | Contig_305 | F: CCACTGAGACGGCTGAAGGT  R: CGAAGCAGTTCCCAAGCAT | (AT)9 | ORF | 277 | 53.8 | CYC02 protein [*Anthurium amnicola*] | 2.00E-05 |
| ZW042 | Contig_331 | F: TCCTCCACCCCAAGAACCT  R: GCCAGGGTCTTGAACACGA | (GCC)5 | ORF | 157 | 58.1 | Farnesylcysteine lyase [*Anthurium amnicola*] | 2.00E-91 |
| ZW043 | Contig_349 | F: TGATTCACTTCGGACGGACA  R: TGGGGCATTTACATCTCTTT | (CT)10 | 3’UTR | 299 | 52.8 | Phosphosulfolactate synthase [*Anthurium amnicola*] | 1.00E-159 |
| ZW044 | Contig_357 | F: CGATTACTTTACCCAGCAGA  R: GGTTTTCTTCACTCCATTCA | (GATC)4A(ATCT)4---(CGGT)4 | 3’UTR | 199 | 48.9 | S-norcoclaurine synthase [*Anthurium amnicola*] | 2.00E-39 |
| ZW045^※^ | Contig_359 | F: GAAGGGGACGGAGAGCAGA  R: CCAGGAACGCAATACTCAT | (GGA)5 | 5’UTR | 238 | 57.1 | Metal transporter Nramp2 [*Anthurium amnicola*] | 1.00E-179 |
| ZW046^※^ | Contig_383 | F: CGACGCCTACCTCCTCAAGA  R: ACGCCCCCTCCCATACATACT | (GCG)6 | ORF | 152 | 54.9 | MLP-like protein 423 [*Anthurium amnicola*] | 1.00E-72 |
| ZW047^※^ | Contig_392 | F: GACGAGTAGATTTAGGCAGA  R: GTAGCACAAGGAAACCATTA | (AG)14 | 5’UTR | 289 | 51.0 | None | None |
| ZW048 | Contig_404 | F: GATGAATAGCAGTCCTCAAGTA  R: CAATAAGACCAGAGTTTCCCAA | (TTCCT)5 | 5’UTR | 260 | 50.5 | unnamed protein product [*Vitis vinifera*] | 5.00E-62 |
| ZW049 | Contig_409 | F: TCACCCACCCATTCCTTCA  R: TTGGTGCCCAGAAGTGAAT | (AG)13A(GAGC)4 | 5’UTR | 215 | 56.3 | Brain protein 44 [*Anthurium amnicola*] | 2.00E-63 |
| ZW050 | Contig_426 | F: TGCTCGGCGGCTTCTTTCT  R: CCCTCTGGAACCTTCTACG | (GCAGGA)4 | 5’UTR | 250 | 57.0 | PREDICTED: 40S ribosomal protein S15 [*Phoenix dactylifera*] | 9.00E-94 |
| ZW051 | Contig_447 | F: CACTATCATCACCAACGCCT  R: CTGATTTGTCTTTCCCTCCA | (TC)13---(CT)13 | ORF | 195 | 54.3 | Calpain-13 [*Anthurium amnicola*] | 2.00E-98 |
| ZW052 | Contig_448 | F: AAGTCTTTCCGTAGCAGTGT  R: ACTTCTATCCGGAGCTCGCT | (GA)16 | ORF | 193 | 52.2 | Polyribonucleotide 5'-hydroxyl-kinase Clp1 [*Anthurium amnicola*] | 8.00E-152 |
| ZW053 | Contig_460 | F: GACCACCCCGAGTCCAAGAA  R: CGCACCCGTCATCAATCAT | (GGCT)4 | 3’UTR | 156 | 54.6 | copper/zinc binding superoxide dismutase [*Paraphaeosphaeria sporulosa*] | 2.00E-95 |
| ZW054 | Contig_491 | F: CGCCAAGGAAGTGGAGACA  R: AGGGTCCGTAATTGCAGCAT | (TGG)5 | ORF | 191 | 56.8 | None | None |
| ZW055 | Contig_517 | F: TCTCAGTTGTCCACCAGCA  R: CTCAGCCTCGCACAGCATT | (TGG)5 | 5’UTR | 167 | 57.9 | Bifunctional inhibitor/plant lipid transfer protein/seed storage helical domain-containing protein [*Cynara cardunculus* var. *scolymus*] | 3.00E-18 |
| ZW056 | Contig_519 | F: GCTCCTGCGGTTTCCTACT  R: ACCCATCACCCCTGTCCTT | (TGC)5 | ORF | 288 | 58.9 | hypothetical protein L484_017734 [*Morus notabilis*] | 2.00E-07 |
| ZW057 | Contig_519 | F: GGGTTCTTGAAAGGATGCT  R: GGCTAATGAACAATCACCT | (AT)10 | 5’UTR | 258 | 50.0 | hypothetical protein L484_017734 [*Morus notabilis*] | 2.00E-07 |
| ZW058 | Contig_554 | F: CCTTCTTGGATGAGATTGT  R: CGACAAAACTATCACTTCCA | (TTTTCG)3 | 5’UTR | 121 | 45.5 | putative hypothetical protein [*Wolffia arrhiza*] | 5.00E-28 |
| ZW059 | Contig_559 | F: AGGAGTAGAGTTGGGTGGA  R: CTTGAGAAGGGAGGATAAAACT | (AG)9 | ORF | 150 | 51.2 | PREDICTED: universal stress protein A-like protein [*Arachis duranensis*] | 1.00E-18 |
| ZW060^※^ | Contig_589 | F: TCAGTCCTCCTCTTCCCCAT  R: GCGTCTTCAGGTCCTTGTAGT | (AGAGGA)4 | 5’UTR | 220 | 56.3 | PREDICTED: NADH dehydrogenase [ubiquinone] 1 alpha subcomplex subunit 2 [*Phoenix dactylifera*] | 1.00E-46 |
| ZW061 | Contig_604 | F: CCTCCTCAAGCGTGGCAAGAT  R: GCAGGTGAAGGCGGCGTA | (CCA)5 | ORF | 145 | 58.0 | hypothetical protein g.65374 [*Anthurium amnicola*] | 4.00E-83 |
| ZW062^※^ | Contig_607 | F: TACTGCAAGGACACCGAAA  R: AATAAGGTATGCCCCCACA | (GC)7 | ORF | 189 | 54.4 | putative xyloglucan endotransglucosylase/hydrolase protein 8 [*Anthurium amnicola*] | 8.00E-74 |
| ZW063 | Contig_615 | F: GTGAACTCCTGAAGGTCTGGTA  R: GACGACTTCACAGCAATCCA | (CCTTT)3 | 5’UTR | 209 | 50.7 | Charged multivesicular body protein 1 [*Anthurium amnicola*] | 1.00E-116 |
| ZW064 | Contig_623 | F: TCTCCCCGCTCACTCATAC  R: AGCCGAAGAACTTGGACCT | (GA)8 | ORF | 152 | 56.6 | Chlorophyll a-b binding protein 6A, chloroplastic [*Anthurium amnicola*] | 5.00E-153 |
| ZW065 | Contig_650 | F: ACCTTTGCCCTGCTGTGAT  R: GGCTGGGGTTCCTTGTGTA | (CTG)6 | ORF | 261 | 59.1 | Lipid transfer protein [*Medicago truncatula*] | 2.00E-20 |
| ZW066 | Contig_676 | F: AGCAGACGAGGTGGCTCTGT  R: CCTGAAGTGGCATCGCAAT | (TCC)5 | 5’UTR | 301 | 58.3 | UPF0548 protein At2g17695 [*Anthurium amnicola*] | 3.00E-68 |
| ZW067 | Contig_680 | F: TCTCTTCCTCTTCATCGCCAT  R: GAGAATGGAGGGGTTCGGAT | (TCGCCG)5 | ORF | 268 | 58.4 | L-ascorbate peroxidase T, chloroplastic [*Anthurium amnicola*] | 5.00E-10 |
| ZW068 | Contig_687 | F: GAGTGCGGTGGTCTCTTTT  R: ATGGTCCGTCTGGTAGTCT | (GGA)5 | 5’UTR | 126 | 53.2 | Mitochondrial outer membrane protein porin [*Anthurium amnicola*] | 3.00E-148 |
| ZW069 | Contig_702 | F: TACTTCCACAAGCTCCGCAA  R: TCCGAACTGAGTCACGTCCA | (AGGCCA)3 | ORF | 141 | 57.8 | 60S ribosomal protein L27a-3 [*Anthurium amnicola*] | 6.00E-33 |
| ZW070 | Contig_703 | ATAGAGGACCACGAGGCGGA | (CT)7 | ORF | 141 | 54.3 | Aspartic proteinase [*Anthurium amnicola*] | 2.00E-29 |
| ZW071^※^ | Contig_707 | F: ACCAGCAGGTACTCCACAT  R: CGAACCAGAACGTCCACGA | (AGG)5 | 5’UTR | 236 | 57.2 | Eukaryotic translation initiation factor 4E-1 [*Anthurium amnicola*] | 2.00E-120 |
| ZW072 | Contig_726 | F: TGCTCCTGCGTCGTCTACC  R: ACACGGGACTACACCAACCA | (TA)7 | ORF | 270 | 51.8 | Aryl hydrocarbon receptor nuclear translocator [*Anthurium amnicola*] | 7.00E-10 |
| ZW073 | Contig_744 | F: TGTGGGAGACGCTGGAGGA  R: CGGAGGAAGCCCGAGAAAA | (TCGCCG)3 | ORF | 121 | 58.8 | Membrane steroid-binding protein 2 [*Anthurium amnicola*] | 1.00E-78 |
| ZW074 | Contig_759 | F: TTGCAGCAATGTGGGGACGT  R: AGCAAGGGGCGAGAGGATAT | (GAA)5 | 5’UTR | 230 | 53.6 | Mannose-1-phosphate guanyltransferase alpha [*Anthurium amnicola*] | 2.00E-91 |
| ZW075 | Contig_761 | F: GACGCCTACCTCCTCAAGA  R: TTACACCATAAGCAAGCCA | (GCG)6 | 3’UTR | 101 | 53.9 | MLP-like protein 423 [*Anthurium amnicola*] | 9.00E-78 |
| ZW076^※^ | Contig_776 | F: CTGAGCAACTACAGCCGAT  R: TATTACGCAGCCCAAACTA | (GCT)5 | ORF | 242 | 50.9 | Laminin subunit gamma-1 [*Anthurium amnicola*] | 1.00E-45 |
| ZW077 | Contig_781 | F: TGGTGGCTGGCGGTGAGAT  R: AGAGGCACCTTCCACGGCAA | (AG)7 | ORF | 229 | 57.6 | None | None |
| ZW078 | Contig_793 | F: GACTTTCCTCTACCCCTTCCA  R: TGCTCTGTTTTGACTTGCCA | (GATGAG)3 | ORF | 230 | 55.7 | PREDICTED: nascent polypeptide-associated complex subunit alpha-like protein 1 [*Phoenix dactylifera*] | 2.00E-90 |
| ZW079 | Contig_801 | F: ACGCATTAGATTTAGACCGA  R: TATGACGAGGATGACGAAGA | (TC)11---(CAC)6 | ORF | 357 | 55.0 | Protein NDR1 [*Anthurium amnicola*] | 3.00E-68 |
| ZW080^※^ | AJ699564 | F: AATGTGCTGGTTCAGATGT  R: CTGGAGTTTTGGTTGATAGT | (GCA)8G(CAA)15 | ORF | 266 | 49.7 | Two-component response regulator ARR2 [*Anthurium amnicola*] | 4.00E-52 |
| ZW081 | AJ699657 | F: GGAGGAAGAGATGAGACGAA  R: CCCGCCCTGTAAAAGATT | (CAG)5 | 5’UTR | 213 | 51.9 | hypothetical protein SELMODRAFT_267228 [*Selaginella moellendorffii*] | 1.00E-04 |
| ZW082 | AJ699753 | F: GAACCACCCTTTCGTCCTT  R: GATGTAAACCCCTACTCGGA | (gaaat)3 | 5’UTR | 184 | 49.3 | None | None |
| ZW083 | AJ699809 | F: CTGTTCTGACCACTGTATTT  R: TCCTCTCCTCCTATTCTATT | (aga)6 | ORF | 205 | 49 | None | None |
| ZW084 | AJ699841 | F: GGACAGGTCTCTGAGCGTGGAA  R: TGAACGGGAGGGCGAAGTAA | (agaag)3 | ORF | 257 | 56.6 | GDSL esterase/lipase At5g45960 [*Anthurium amnicola*] | 1.00E-114 |
| ZW085 | AJ699883 | F: TTCCCCTCCTCACCCACCCATT  R: GGATCTTCGCCGCTGCCATA | (ag)15 | 5’UTR | 170 | 59.7 | Brain protein 44 [*Anthurium amnicola*] | 1.00E-63 |
| ZW086 | AJ699974 | F: AGCAGCCTGACCCATTAGT  R: TGCGAAAGATTCCTCACTT | (tgag)4 | 3’UTR | 151 | 48.3 | Dof zinc finger protein DOF5.2 [*Anthurium amnicola*] | 9.00E-53 |
| ZW087 | AJ699979 | F: ATTTATGCGGACGCCATATA  R: GCACAAGGAAACCATTACA | (ag)7 | ORF | 272 | 57.1 | None | None |
| ZW088 | AJ700062 | F: TACTAACACTATCCAGGGT  R: TATTACATCGAGGTGACACT | (ag)8 | 3’UTR | 149 | 47.8 | None | None |
| ZW089 | AJ700063 | F: TGCTGTTGTTAAGGGTCGT  R: CACTCTTCTTTTCATCGGA | (acccc)3 | 5’UTR | 200 | 53.3 | Mitochondria fission 1 protein [*Anthurium amnicola*] | 1.00E-77 |
| ZW090 | AJ700113 | F: GGGTCTGGACTAGCCTGTA  R: TACGAGTAGCAGTCAGCGA | (tc)7 | ORF | 153 | 53.9 | Cysteine proteinase 1 [*Anthurium amnicola*] | 3.00E-91 |
| ZW091 | AJ700139 | F: TCCTGAATTGATGAGGCTA  R: CAGATAACAATGTACAGATCCT | (aaaag)4 | 3’UTR | 224 | 47.1 | Mitochondrial substrate carrier family protein Q [*Anthurium amnicola*] | 2.00E-56 |
| ZW092 | AJ700167 | F: GAGCCGAGGAGGAAGAAGT  R: ATGTGAAGGCAGGCAGAGA | (tc)7 | ORF | 251 | 57.3 | PREDICTED: 15.4 kDa class V heat shock protein [*Musa acuminata* subsp. *malaccensis*] | 8.00E-08 |
| ZW093^※^ | AJ700207 | F: CTCCTCTCCTCAGTTAGTA  R: GGTATACATGCTGGGTACA | (at)7 | ORF | 157 | 46.6 | Trypsin/chymotrypsin inhibitor [*Anthurium amnicola*] | 2.00E-15 |
| ZW094 | AJ700389 | F: GGTATGCCCAGTGGTGTTGT  R: GCTGCGCAGAGATAGAGAAGA | (tct)5 | 5’UTR | 150 | 54.8 | PREDICTED: superoxide dismutase [Cu-Zn] 2 [*Musa acuminata* subsp. *malaccensis*] | 1.00E-81 |
| ZW095^※^ | AJ700448 | F: CAGTTCATCTACGGCTCCT  R: GGCACTTGGAAACAATTAGT | (tc)8 | 5’UTR | 186 | 53 | PREDICTED: LOW QUALITY PROTEIN: probable pectinesterase/pectinesterase inhibitor 34 [*Phoenix dactylifera*] | 4.00E-59 |
| ZW096^※^ | AJ700498 | F: GAGAAGGCACCGAGGAAAA  R: GTGTCGAACAGCGAGAACG | (ga)9 | ORF | 127 | 57.1 | hypothetical protein GLYMA_01G211700 [*Glycine max*] | 1.00E-84 |
| ZW097 | AJ700498 | F: GCAAAATCCGCTACGAAGA  R: CCACCCTAAAACCAAATCAA | (aatttcc)3 | ORF | 228 | 52.3 | hypothetical protein GLYMA_01G211700 [*Glycine max*] | 1.00E-84 |
| ZW098^※^ | AJ700524 | F: GTAGGGATGGCGGCAATGGA  R: AGGCGGTTGAGGTTGTGGGT | (ggc)5 | ORF | 191 | 60.6 | 26S protease regulatory subunit 8 A [*Anthurium amnicola*] | 2.00E-167 |
| ZW099 | AJ700534 | F: AGTCTTAGGGAACCAACCA  R: GAATCCTCAAAGAGGCC | (ctc)6 | ORF | 108 | 52.8 | PREDICTED: oxygen-evolving enhancer protein 1, chloroplastic [*Eucalyptus grandis*] | 5.00E-147 |
| ZW100 | AJ700607 | F: TGTCCTCAGTGTAACCAAT  R: GCACAGAGAAGAATCCATA | (gtttt)4 | 3’UTR | 148 | 44.7 | Zinc finger BED domain-containing protein 1 [*Anthurium amnicola*] | 7.00E-98 |
| ZW101^※^ | AJ700640 | F: CCCAGATGGAACTGCTCAA  R: AGGCGAGGGAGGACTTGTA | (gca)6 | ORF | 268 | 55.5 | None | None |
| ZW102^※^ | AJ700653 | F: CCCAAATCCCAGTTTCTTA  R: CCATTCCACTTGAATCTGAA | (ttcat)3 | 5’UTR | 143 | 46.3 | None | None |
| ZW103 | AJ700703 | F: GAAGCAGGGAGTGAGGAGA  R: ACAGGTAGCACCAGCATTGA | (cgc)7 | 5’UTR | 276 | 55.8 | Amidase 1 [*Anthurium amnicola*] | 2.00E-126 |
| ZW104 | AJ700812 | F: TGTTACTGTTTCCTCCTCA  R: CTTGAAAGACTATCCCTGA | (ca)8 | ORF | 212 | 48.7 | Exostosin-2 [*Anthurium amnicola*] | 4.00E-103 |
| ZW105 | AJ700855 | F: CAGACACTTGAAGGGGCTA  R: CGAGACCAAATGACGAGTAA | (ta)9 | ORF | 268 | 48.6 | None | None |
| ZW106 | AJ700922 | F: CTTCTTCTTCTTCCTGTGTT  R: CTCATAGAGCCCCCCCTTT | (ag)16 | 5’UTR | 112 | 49.4 | BI1-like protein [*Anthurium amnicola*] | 3.00E-96 |
| ZW107 | AJ700992 | F: GGATCATGCTGCCAAGATAAAGA  R: TCCCTTTGGAGTAGCCATTCA | (TC)7 | ORF | 219 | 53.5 | Oxygen-evolving enhancer protein 3-2, chloroplastic [*Anthurium amnicola*] | 1.00E-17 |
| ZW108 | AJ701018 | F: CCTAGTTCCCGAGGAGTACA  R: AGGATGGGTGTAGAAGGGA | (aaaat)7 | 5’UTR | 178 | 54.9 | unnamed protein product [*Coffea canephora*] | 1.00E-58 |
| ZW109 | AJ701019 | F: TGGCGACCAAGGAAGTTTA  R: AACGCATCTACCAGAACGCT | (ag)9 | ORF | 273 | 55.2 | None | None |
| ZW110 | AJ701048 | F: CCCCATCACATTCTCTGCT  R: AGTTTTGTCACGCCCTCTT | (TC)25 | ORF | 178 | 53.3 | None | None |
| ZW111 | AJ701099 | F: GCTTTTCTCTTACCTCGCA  R: TTCAGTTCTTCAGCGTCCT | (gaa)8 | ORF | 272 | 57.6 | PREDICTED: small nuclear ribonucleoprotein E-like isoform X1 [*Nelumbo nucifera*] | 6.00E-50 |
| ZW112 | AJ701156 | F: ATCTCCACCGCTGAGCAGTT  R: CTCCTCCACGGGCAAACTAA | (CTG)6 | ORF | 264 | 58.4 | None | None |
| ZW113 | AJ701159 | F: AGGAGATGGAAAATACGGCA  R: CTAGCGACCAATTCAAGAGAA | (ttttc)3 | 3’UTR | 110 | 46.5 | putative calcium-binding protein CML11 [*Anthurium amnicola*] | 1.00E-30 |
| ZW114 | AJ701182 | F: CTGGTGAAGTGGAACGAGA  R: TAGCGGCTGTGGAATAAAA | (tga)5 | ORF | 183 | 52.1 | hypothetical protein ACMD2_07761 [*Ananas comosus*] | 9.00E-40 |
| ZW115 | AJ701207 | F: AAAAATGTGATTAGAGGGG  R: AAAAACCCAAACACCAAAA | (tttttg)3---(tttgt)3 | ORF | 312 | 45.4 | None | None |
| ZW116 | AJ701282 | F: AGACGCTTGTTCCGTGACTTA  R: TTCAATGGTACCTTGGTGCTT | (agcctc)3 | 5’UTR | 213 | 52.3 | Casein kinase I isoform delta-like [*Anthurium amnicola*] | 9.00E-47 |
| ZW117 | AJ701508 | F: CAGAGATTGGAGAGAGCAGA  R: GGATGAAGGTTAAAGATGAGGT | (ggc)5 | ORF | 192 | 56.1 | putative ascorbate-specific transmembrane electron transporter 1 [*Anthurium amnicola*] | 9.00E-126 |
| ZW118 | AJ701520 | F: CCATCCTCACCACTTAAAA  R: GAAGAAAACGAAGAGCAGA | (tcttct)4 | 3’UTR | 137 | 49.5 | phospholipase D, partial [*Triticum turgidum* subsp. *durum*] | 7.00E-71 |
| ZW119 | AJ701579 | F: GAAAGGAGCGAAGGGACAT  R: ACCACGAAACAAACGACGA | (ct)9 | 5’UTR | 196 | 51.4 | None | None |
| ZW120 | AJ701653 | F: GACCATCGTCTCCCACTGA  R: CAGCAAAACCAAGAGACAGAT | (tcc)5 | 3’UTR | 275 | 52.4 | putative GDP-D-mannose pyrophosphorylase [*Amorphophallus konjac*] | 4.00E-09 |
| ZW121 | AJ701698 | F: TTCCCCGAGCAGTCCAACGT  R: AGAGGAGGCAGAAGGAGAGCA | (cct)5 | 5’UTR | 160 | 59.4 | None | None |
| ZW122^※^ | AJ701740 | F: CAGCAAGAAGCCCAAGCCT  R: GGAATCTCTCCAGACAAGAAATCA | (at)16 | ORF | 221 | 50.2 | PREDICTED: uncharacterized protein At1g15400-like [*Cicer arietinum*] | 2.00E-08 |
| ZW123^※^ | AJ701809 | F: TACCTGCCCCACCCACTGTT  R: TGCATCCCCGTAATTCACCT | (cag)6 | 5’UTR | 125 | 54 | PREDICTED: putative clathrin assembly protein At2g01600 [*Musa acuminata* subsp. *malaccensis*] | 2.00E-18 |
| ZW124 | AJ701839 | F: AGAACCCCTCTTCACATAGA  R: CAGAATAAACGAACACCCAT | (agc)5 | ORF | 216 | 52.8 | UDP-galactose/UDP-glucose transporter 4 [*Anthurium amnicola*] | 9.00E-90 |
| ZW125 | AJ701914 | F: TACCCCTACCGGTCCTACT  R: TAGTCATCGGAGCCACATA | (cgg)5 | ORF | 281 | 58.8 | Major capsid protein L1 [*Anthurium amnicola*] | 5.00E-23 |
| ZW126^※^ | AJ701918 | F: CGAGCAGAACATTCAGCAGT  R: CCATCGCAGTAAATCGTAGA | (tg)9 | ORF | 186 | 51.1 | Transcription factor BIM2 [*Anthurium amnicola*] | 1.00E-07 |
| ZW127 | AJ702043 | F: CTCCTCTTTCGCTCCCCTT  R: CCCCTCTCTCCCTTCTTTT | (ctg)5 | ORF | 159 | 52.7 | 3-ketoacyl-CoA synthase 4 [*Anthurium amnicola*] | 7.00E-10 |
| ZW128 | AJ702043 | F: TTACAGGGGCTGACGAAAT  R: TCCATCCACTCCACCAACA | (ctg)6 | ORF | 195 | 54.3 | 3-ketoacyl-CoA synthase 4 [*Anthurium amnicola*] | 7.00E-10 |
| ZW129 | AJ702057 | F: CTAACACCATCAAAAGGTCGT  R: TCTTCTTCGGAACCCTAACA | (tct)6 | 5’UTR | 175 | 54.4 | Histone-lysine N-methyltransferase ATX5 [*Anthurium amnicola*] | 4.00E-44 |
| ZW130 | AJ702151 | F: TGACGCAAGTAACTATTGTAGGT  R: CTCAGGTGATTCCATTTCCA | (ag)17 | 5’UTR | 298 | 50.8 | hypothetical protein g.47289 [*Anthurium amnicola*] | 3.00E-15 |
| ZW131 | AJ702156 | F: TGAGGTCCATCCTGTCGTGT  R: CATCTGTGCCACTGACCAAAA | (aaggg)3---(tc)9 | ORF | 158 | 50.3 | None | None |
| ZW132^※^ | AJ702276 | F: CCATCTTCTCTGAGGGGAAGAA  R: AAGGGAGGCTGCTGTATGCGT | (tcgccg)3 | 5’UTR | 156 | 56.9 | Protein YIPF1 [*Anthurium amnicola*] | 2.00E-71 |
| ZW133 | AJ702357 | F: CTCTGTGTCGCTTCTCTTGT  R: CGCAGGGAAACCATTACAT | (ag)8 | ORF | 124 | 50.6 | None | None |
| ZW134 | AJ702432 | F: TGCTGAGTGGGGTGAAAGT  R: CCATACCAGAAAAGAGCGAAT | (tc)9 | ORF | 271 | 53.4 | None | None |
| ZW135 | AJ702535 | F: TCTCCCTCCAAGGAAGCGT  R: CTCGTCCCCGTAGTACACCT | (ag)7 | ORF | 120 | 54.3 | PREDICTED: uncharacterized protein LOC105052616 [*Elaeis guineensis*] | 8.00E-66 |
| ZW136 | AJ702581 | F: TGGGCAAGATGGACGAGTA  R: TGGATGTGGTGTTGGTGGA | (ccacc)3 | ORF | 202 | 55.2 | transcription initiation factor IID, TAF10 subunit [*Aureobasidium pullulans* EXF-150] | 1.00E-82 |
| ZW137 | AJ702613 | F: CACCATTACCCGCTTCTCT  R: CCAGTTCTGACCTCTCCGA | (cggtct)4 | ORF | 150 | 53.8 | None | None |
| ZW138 | AJ702669 | F: GACGCCTACCTCCTCAAGAA  R: GCCCCCTCCCATACATACT | (gcg)6 | ORF | 149 | 53.5 | MLP-like protein 423 [*Anthurium amnicola*] | 8.00E-04 |
| ZW139 | AJ702698 | F: TTTACTACCCCGTCCCTCGT  R: GCAACACATAAGCCCATACT | (GA)26 | ORF | 289 | 54.7 | hypothetical protein MANES_18G044900 [*Manihot esculenta*] | 7.00E-30 |
| ZW140^※^ | AJ702737 | F: TGGTCGGCCGCTCCTTCT  R: CCCTCTGCCTGTCCAACTCCT | (cca)6 | ORF | 234 | 60.7 | None | None |
| ZW141 | AJ702739 | F: CTGCTACGGTGGTTCTTTA  R: TTTCGGACTTGGACACATA | (tc)11 | 3’UTR | 274 | 50.6 | None | None |
| ZW142 | AJ702779 | F: CCTGGGAACTACCTTGGACT  R: GGGAGCGGTCTCTTCTTGA | (tgg)7 | ORF | 209 | 54.9 | putative RNA-binding protein C25G10.01 [*Anthurium amnicola*] | 6.00E-36 |
| ZW143 | AJ702798 | F: AGATCTCATTCACGTTT  R: AGCAACTCTCAATTTAA | (TA)8 | 5’UTR | 130 | 42.5 | Proton-coupled amino acid transporter 3 [*Anthurium amnicola*] | 2.00E-36 |
| ZW144 | AJ702925 | F: TCGGCGGACCATCTCCAAGA  R: CGACGCCCTTGAGGTAGCCATT | (acccta)3 | ORF | 204 | 60.6 | PREDICTED: 60S ribosomal protein L8-like [*Jatropha curcas*] | 1.00E-166 |
| ZW145 | AJ702978 | F: TACCGGTCCGGATTCCCTT  R: GTTGGAGTTGTCCCCGAAGA | (tc)8 | ORF | 260 | 58.1 | Copper transporter 5 [*Anthurium amnicola*] | 4.00E-20 |
| ZW146^※^ | AJ703002 | F: CGCCCAAGAAGTGGGAGAGT  R: AAGCCAGGGAACCGGAGAGA | (gaa)6 | 5’UTR | 116 | 56.8 | Ferredoxin-thioredoxin reductase catalytic chain, chloroplastic [*Anthurium amnicola*] | 3.00E-84 |
| ZW147 | AJ703007 | F: TCTCAATTCGCGACGTTAT  R: CTCTCCCTGAAGATGCTGTT | (ct)9 | 3’UTR | 252 | 54.5 | ribonucleoprotein, chloroplastic [*Anthurium amnicola*] | 6.00E-49 |
| ZW148 | AJ703153 | F: ACGCGTCCGTGCACCCA  R: CGCCGTCCCTAATCTTTT | (gaaaga)3 | 5’UTR | 110 | 53.1 | PREDICTED: uncharacterized protein LOC105043846 isoform X2 [*Elaeis guineensis*] | 6.00E-09 |
| ZW149 | AJ703162 | F: CATCCACCAAAGTTGACAT  R: TTCTCCTGAGGTAGAGCCT | (aga)5 | ORF | 148 | 49.2 | 60S ribosomal protein L6 [*Anthurium amnicola*] | 2.00E-89 |
| ZW150 | AJ703251 | F: TGGAGGTACTGGTCTAGGA  R: ATATACGCTCTTCAGGGTGA | (ggt)5 | ORF | 258 | 56.3 | None | None |
| ZW151 | AJ703251 | F: CGGCTTCACCATAAATAGT  R: TAAAGGCACATCACACAAA | (at)11 | 3’UTR | 198 | 45.9 | None | None |
| ZW152 | AJ703277 | F: ACCCCTTCAACTGGAGCGA  R: AGCGGTGGGGAGAGTAGTTGT | (caa)6---(aac)7 | ORF | 195 | 56.2 | hypothetical protein ZOSMA_2G03350 [*Zostera marina*] | 2.00E-15 |
| ZW153 | AJ703277 | F: GTCTCATGTACGTGATGCTGT  R: CTGCTCTTTCCTCTGTCTGTT | (aaaga)3 | 3’UTR | 188 | 52.6 | hypothetical protein ZOSMA_2G03350 [*Zostera marina*] | 2.00E-15 |
| ZW154 | AJ703288 | F: GCCATCAGAATCACAGACCT  R: ATGCTCCACAGAGTGCCTT | (aag)5 | 5’UTR | 122 | 51.4 | PHD finger protein ALFIN-LIKE 5 [*Anthurium amnicola*] | 3.00E-72 |
| ZW155 | AJ703307 | F: CCGCAGCAGTCTGTGTGAT  R: AGGGGAGAATAAGGCGTTGT | (agggg)3 | 5’UTR | 242 | 55.7 | Trans-2,3-enoyl-CoA reductase [*Anthurium amnicola*] | 1.00E-137 |
| ZW156 | AJ703312 | F: ATGTGGGGTAGTGGTCAAT  R: GCCAATCTGTTATATAGTGT | (ttttct)3 | 5’UTR | 202 | 46.5 | PREDICTED: uncharacterized protein LOC103996648 [*Musa acuminata* subsp. *malaccensis*] | 4.00E-43 |
| ZW157 | AJ703322 | F: AGCGGCGCACAGAGCTTTTA  R: CATCAACCATTTCTTCCCCAA | (ag)13 | 5’UTR | 256 | 59.2 | Endothelin-converting enzyme 2 [*Anthurium amnicola*] | 4.00E-130 |
| ZW158^※^ | AJ703328 | F: CAATAGCGAAATCGTCAACA  R: TCACACGGTCATAAAGCAT | (tta)8 | ORF | 203 | 49.4 | None | None |
| ZW159 | AJ703399 | F: CGCTTCTTTCTCGCTCGGA  R: CGGCGGCTTTTCTGGTTTT | (gcta)4 | 3’UTR | 290 | 59.2 | Acireductone dioxygenase [*Anthurium amnicola*] | 7.00E-18 |
| ZW160 | AJ703468 | F: GAGGGGAGGACTCGTCAAGA  R: TCATCCTTTGCTGGCTCGT | (ctctgg)3 | ORF | 197 | 58.4 | xyloglucan endotransglycosylase XET2 [*Asparagus officinalis*] | 4.00E-88 |
| ZW161 | AJ703501 | F: CAAGCCAGCAAGCAGGAGGA  R: CTTTGATGCCCACACCCCAAT | (ag)14 | ORF | 139 | 55.6 | PREDICTED: heavy metal-associated isoprenylated plant protein 26-like [*Elaeis guineensis*] | 2.00E-35 |
| ZW162 | AJ703521 | F: AAGGTCTTCCCCCCCAGGTA  R: GCGTTAGCACCGACCGATTT | (ctt)7 | 5’UTR | 111 | 51.3 | Hydrophobic protein LTI6A [*Cajanus cajan*] | 2.00E-08 |
| ZW163 | AJ703526 | F: CAACAGCAACAGCAACAGCA  R: TTGGTGCCTGCTGCCTAAT | (cag)5 | 3’UTR | 286 | 54 | hypothetical protein g.24782 [*Anthurium amnicola*] | 2.00E-21 |
| ZW164 | AJ703526 | F: TATGCCAGAGAAGGATGAA  R: TATGCCAGAGAAGGATGAA | (catcaa)6---(caacag)4 | 3’UTR | 277 | 51.3 | hypothetical protein g.24782 [*Anthurium amnicola*] | 2.00E-21 |
| ZW165^※^ | AJ703609 | F: TCGCCTGGGTGTATTATCT  R: TTGGGTGGTTTCTATTGCT | (TC)12 | ORF | 198 | 50.4 | Syntaxin-binding protein 5 [*Anthurium amnicola*] | 3.00E-48 |
| ZW166 | AJ703611 | F: GGAAGAAGACGAGAGCAAGA  R: CCGACTTTGATACTTCAGAGAT | (TCC)5 | ORF | 328 | 58.1 | UPF0250 protein mma_3250 [*Anthurium amnicola*] | 1.00E-82 |

Note: “※” indicate polymorphic EST-SSR loci across the 12 cultivars of colored calla lily.
