# Supplementary material for: Assessing Genetic Diversity and Population Differentiation of Colored Calla Lily (Zantedeschia Hybrid) for an Efficient Breeding Program
Source: Genes (Basel). 2017 Jun 21;8(6):168. doi: 10.3390/genes8060168 (PMC5485532; doi:10.3390/genes8060168)
Supplement: Supplementary file 1 [file genes-08-00168-s001.zip › Table S3.docx]

**Table S2.** KEGG annotation and its corresponding EC number of 182 SSR-containing unigenes of white calla lily.

| **Pathway** | **Sequences in Pathway** | **Enzyme and its ID** | **Sequences of** **Enzyme** | **Sequences** | **Pathway ID** |
| --- | --- | --- | --- | --- | --- |
| Biosynthesis of antibiotics | 2 | ec:5.1.1.7 - epimerase | 2 | Contig_280 | map01130 |
| Biosynthesis of antibiotics | 2 | ec:4.6.1.12 - 2,4-cyclodiphosphate synthase | 2 | AJ700908 | map01130 |
| Phenylpropanoid biosynthesis | 2 | ec:1.11.1.7 - lactoperoxidase | 1 | Contig_680, AJ701681 | map00940 |
| Lysine biosynthesis | 1 | ec:5.1.1.7 - epimerase | 1 | Contig_280 | map00300 |
| Amino sugar and nucleotide sugar metabolism | 1 | ec:2.7.7.13 - guanylyltransferase | 1 | Contig_759 | map00520 |
| Glycerophospholipid metabolism | 1 | ec:3.1.4.4 - D | 1 | AJ701520 | map00564 |
| Methane metabolism | 1 | ec:4.4.1.19 - synthase | 1 | Contig_349 | map00680 |
| D-Glutamine and D-glutamate metabolism | 1 | ec:5.1.1.10 - racemase | 1 | Contig_280 | map00471 |
| Purine metabolism | 1 | ec:3.6.1.3 - adenylpyrophosphatase | 2 | Contig_46 | map00230 |
| Purine metabolism | 1 | ec:3.6.1.15 - phosphatase | 2 | Contig_46 | map00230 |
| D-Arginine and D-ornithine metabolism | 1 | ec:5.1.1.10 - racemase | 1 | Contig_280 | map00472 |
| Pentose and glucuronate interconversions | 1 | ec:3.1.1.11 - pectin demethoxylase | 1 | AJ700448 | map00040 |
| Fructose and mannose metabolism | 1 | ec:2.7.7.13 - guanylyltransferase | 1 | Contig_759 | map00051 |
| Glycine, serine and threonine metabolism | 1 | ec:5.1.1.10 - racemase | 1 | Contig_280 | map00260 |
| Terpenoid backbone biosynthesis | 1 | ec:4.6.1.12 - 2,4-cyclodiphosphate synthase | 1 | AJ700908 | map00900 |
| Thiamine metabolism | 1 | ec:3.6.1.15 - phosphatase | 1 | Contig_46 | map00730 |
| Drug metabolism - other enzymes | 1 | ec:3.1.1.1 - ali-esterase | 1 | AJ700448 | map00983 |
| Ether lipid metabolism | 1 | ec:3.1.4.4 - D | 1 | AJ701520 | map00565 |
| Starch and sucrose metabolism | 1 | ec:3.1.1.11 - pectin demethoxylase | 1 | AJ700448 | map00500 |
| Cysteine and methionine metabolism | 1 | ec:5.1.1.10 - racemase | 1 | Contig_280 | map00270 |
