# Supplementary material for: Assessing Genetic Diversity and Population Differentiation of Colored Calla Lily (Zantedeschia Hybrid) for an Efficient Breeding Program
Source: Genes (Basel). 2017 Jun 21;8(6):168. doi: 10.3390/genes8060168 (PMC5485532; doi:10.3390/genes8060168)
Supplement: Supplementary file 1 [file genes-08-00168-s001.zip › Table S4.docx]

**Table S4.** Pairwise population Fst Values (*P* < 0.001) between inferred four sub-groups of colored calla lily.

|  | **G-Ia** | **G-Ib** | **G-IIa** | **G-IIb** |
| --- | --- | --- | --- | --- |
| G-Ia | 0.000 | 0.001 | 0.001 | 0.001 |
| G-Ib | 0.179 | 0.000 | 0.001 | 0.001 |
| G-IIa | 0.234 | 0.179 | 0.000 | 0.001 |
| G-IIb | 0.281 | 0.234 | 0.142 | 0.000 |

Note: *Fst* Values below diagonal. Probability values based on 999 permutations are shown above diagonal.
