# Supplementary material for: Assessing Genetic Diversity and Population Differentiation of Colored Calla Lily (Zantedeschia Hybrid) for an Efficient Breeding Program
Source: Genes (Basel). 2017 Jun 21;8(6):168. doi: 10.3390/genes8060168 (PMC5485532; doi:10.3390/genes8060168)
Supplement: Supplementary file 1 [file genes-08-00168-s001.zip › Figure S1.docx]

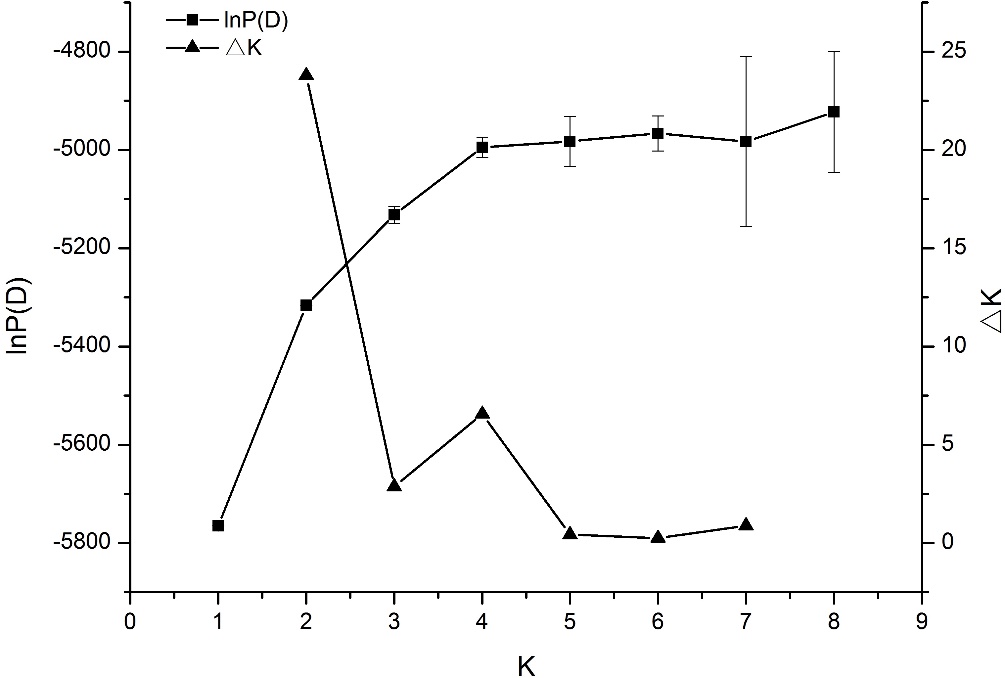


**Figure S1.** Estimates of the posterior probability of the data at a given K for 117 colored calla lily accessions. LnP(D) obtained in Structure analysis and an ad hoc quantity ΔK based on the second-order rate of change of the likelihood function with the respect to K computed for 31 EST-SSR loci.
